# Destination choices during internal temporary migration: Evidence from northern Bangladesh

**DOI:** 10.1371/journal.pone.0352665

**Published:** 2026-07-01

**Authors:** Md. Sohel Rana, Amy Faye

**Affiliations:** Center for Development Research (ZEF), University of Bonn, Bonn, Germany; Sun Yat-Sen University, CHINA

## Abstract

While migration to urban areas is often associated with higher wage opportunities, it remains unclear why many rural poor prefer rural destinations, particularly for temporary migration. We investigate this in northern rural Bangladesh, where temporary migration is common, by analyzing both the determinants of rural versus urban temporary migration and their income effects. We examine temporary migrants’ destination choices, accounting for their self-selection into migration, and address endogeneity when estimating the income effects of these choices. Despite recent findings that perceived income gains tend to be higher from rural destinations, our findings show that rural destinations are not necessarily better than urban ones at increasing actual total household income. In fact, remittances from rural-bound temporary migration are lower than those from urban-bound migration. However, our results also suggest that rural-bound migration is not primarily driven by remittance maximization. Instead, constrained by household obligations, temporary migrants often prefer rural destinations because they offer greater income-to-cost ratio, permit continued engagement in origin-based labor activities, and hence facilitate household risk diversification while reducing time away from home.

## Introduction

Globally, around 682 million people live in extreme poverty, of whom, around 75% reside in rural agrarian societies where they inevitably face livelihood fluctuations and seasonal hunger during agricultural lean periods [1,2]. In northern Bangladesh, for example, a 2–3 months lean period occurs twice a year between the planting and harvesting of staple crops, affecting around seven million rural poor from around two million agricultural labor-dependent households [3–6]. Studies have identified temporary migration as a common strategy for the rural poor to cope with income seasonality and seasonal hunger during these lean periods, when on-farm wage opportunities drastically drop in the origin villages [2,4,5,7–9].

Existing migration theories predict that such income-driven migration, originating from the low-productive rural agricultural sector, flows toward higher-paying modern sectors located in urban areas [10–13]. Consequently, the assumption of temporary migration from rural to urban destinations is common in the existing literature [4,7,14–20]. However, studies also find that around 65% of temporary migrants from northern rural Bangladesh migrate to other rural destinations [21], despite the higher wage opportunities in Bangladeshi cities [4,17]. Similar rural-to-rural temporary migration is also common in neighbouring Myanmar and India [22,23]. This raises a fundamental question as to why do many temporary migrants prefer rural over urban destinations, a topic poorly understood in the existing literature, which we address in this paper.

Several studies have examined destination choices for internal migration, but mainly focusing on permanent or longer-term migration types, which differs in nature from temporary migration [24–28]. To our knowledge, only one study [29] has examined destination choices for temporary migration, employing an exploratory qualitative methodology with a relatively small sample size. This study highlights that the timing and duration of lean periods vary across regions due to differences in cropping patterns, thereby facilitating rural-to-rural migration. Regarding destination decision-making, the study emphasizes the importance of migrants’ individual characteristics, their networks, and perceptions about destinations in shaping their choices. Notably, this study finds greater income gain perception for rural relative to urban destination choices, questioning the common assumption that urban destinations offer greater gains.

Yet perceived gains may not necessarily correspond to actual income effects. Destination choices may instead reflect differences in migration costs, remittance patterns, and households’ need to balance migration with origin-based livelihood activities, aspects that remain unclear in the existing literature. By examining both the determinants and income effects of temporary rural versus urban migration, we investigate whether destination choices are primarily driven by realized economic returns or by broader household risk-diversification strategies.

We employ a quantitative methodology to achieve this. Specifically, we pursue two objectives: 1) identifying factors explaining temporary migration decisions to rural versus urban destinations, and 2) investigating the household-level income effects of such destination choices. We organize this article as follows: the next section presents the sampling, data, conceptual framework, and models used to achieve our research objectives. The empirical results are then presented and discussed. Finally, we conclude the article and outline its policy implications.

## Materials & methods

### Sampling procedure

We employ a multi-stage sampling procedure to select study areas and collect data, following [9]. In the first stage, we purposively select Rangpur Division of Bangladesh–the poorest division in the country, where agricultural seasonality is more pronounced and temporary migration is more common compared to other regions [5,8,30]. Approximately 47% of rural households in this region send migrants temporarily [2], with many migrating to other rural destinations in search of temporary farm jobs [4,21], making this region a compelling case for study.

The Rangpur Division consists of eight districts. Among them, in the second stage, we purposively select the two poorest districts, Dinajpur and Kurigram, which hold the highest proportion of agricultural labor-dependent households that are more vulnerable to agricultural seasonality thus more prone to temporary migration during lean periods [3,8,30]. Dinajpur district comprises of 2,131 villages, while Kurigram has 1,872 villages [31]. In the third stage, we follow stratified random sampling to select a total of 30 villages- 16 from Dinajpur and 14 from Kurigram.

In the fourth stage, we randomly survey households in those villages. Following village selection, we collect household lists for the selected villages from the respective local government offices. The 30 selected villages have a total of 7,441 households. At 99% confidence level and 5% margin of error, a minimum sample of 612 households is required. We randomly select 10% of households from each village, with an additional 6% as replacements in case of non-response. We survey a total of 878 households; sample distribution is presented in Table A1 in the Online Appendix. The surveyed households include approximately 10–14% of total households in each surveyed village.

### Survey data collection

There are two dominant agricultural lean periods in northern Bangladesh: the *Aman* lean in September-November between planting and harvesting the *Aman* seasonal crops, and the *Boro* lean in February-April between planting and harvesting the *Boro* seasonal crops [4,9,32]. The rest of the year are considered normal periods with normalized wage opportunities in the origin villages [9]. We conduct the survey during the *Boro* post-harvest period, June-August 2023, when most temporary migrants are in their villages to harvest the *Boro* seasonal crops and plant the next *Aman* seasonal crops.

The survey was administered with the head of the household who is often the migrant member. We collect key demographic characteristics, e.g., age, gender, education, and occupation, labor participation at the origin, and detailed migration data for every member of the household. At the household level, we collect data about households’ assets, agriculture farming, migrant networks, and employment availability during the lean and normal periods, and seasonal income from farm and non-farm sources. During the survey, we referred to the past 12 months (August 2022- July 2023) for collecting time-variant data such as farming, migration, and income. We also collected individual migrants’ perceptions about destination cities prior to making their first migration.

The survey questionnaire was programmed in SurveyCTO. Before conducting the survey, we obtained informed verbal consent from participants through SurveyCTO, clearly communicating the purpose of the study and emphasizing the voluntary nature of their participation. The consent form is provided in the Online Appendix A. Individuals’ data were analyzed anonymously. Also, the study protocol was reviewed and approved by the research ethics board of the authors’ institution in August 2022.

### Socio-demographic and migration data

Agriculture is the main source of livelihood in the study areas, as in other rural areas of the country. Roughly three-quarters of surveyed households are engaged in crop farming, and around 62% also practice household-level livestock farming as a risk diversification strategy. Small-sized households, averaging four members, are dominant in the region—roughly 60% in our dataset—and typically prefer temporary over longer-term migration, when migration is necessary [9].

In our dataset, 371 households (~42%) did not send any migrants for income. On the other hand, 330 households (~38%) sent exclusively temporary migrants, who migrated for a period of up to three months per episode and actively participated in the origin village’s labor market upon every return. The dataset also contains 150 households (~17%) that sent exclusively longer-term migrants, and 27 households (~3%) sending both types of migrants simultaneously.

The dataset comprises 3,818 individuals from 878 households. Since destination choice is largely influenced by migrants’ individual characteristics [29,33], we utilize the individual members’ dataset. From this dataset, we exclude 44 individuals migrating for immediate non-income purposes, such as pursuing education. Moreover, we remove 981 children aged 14 years or younger, as they rarely migrate for income. After these exclusions, the dataset comprises 2,793 individual members: 385 (~14%) engaged exclusively in temporary migration, 220 (~8%) exclusively in longer-term migration, and 2,188 (~78%) non-migrants. Our dataset shows no participation of women in temporary migration. This likely reflects both the social stigma surrounding this type of migration and the growing availability of garment industry employment, which offers longer-term opportunities for women migrants with relatively better working conditions and social dignity [29,34]. Among our longer-term migrants, only 9% are women, all of whom are employed in the garment sector.

Among the 385 temporary migrants, 259 individuals (~67%) migrated to rural destinations, and 126 individuals (~33%) to urban destinations during their latest migration episode. Following the definition provided by the Bangladesh Bureau of Statistics [35], we classify urban destinations as areas located within the jurisdictions of City Corporations, Municipalities, Sub-district Headquarters (not declared as Municipalities), Cantonment areas, and Growth Centres enlisted by the Local Government Division. All other areas are classified as rural destinations. Additionally, we collect information on the type of work undertaken by migrants at their destinations, which we use to validate destination types and to further differentiate between rural and urban destinations when migrants are unable to identify the destination type.

### Conceptual framework and model specifications

**Fig 1:**
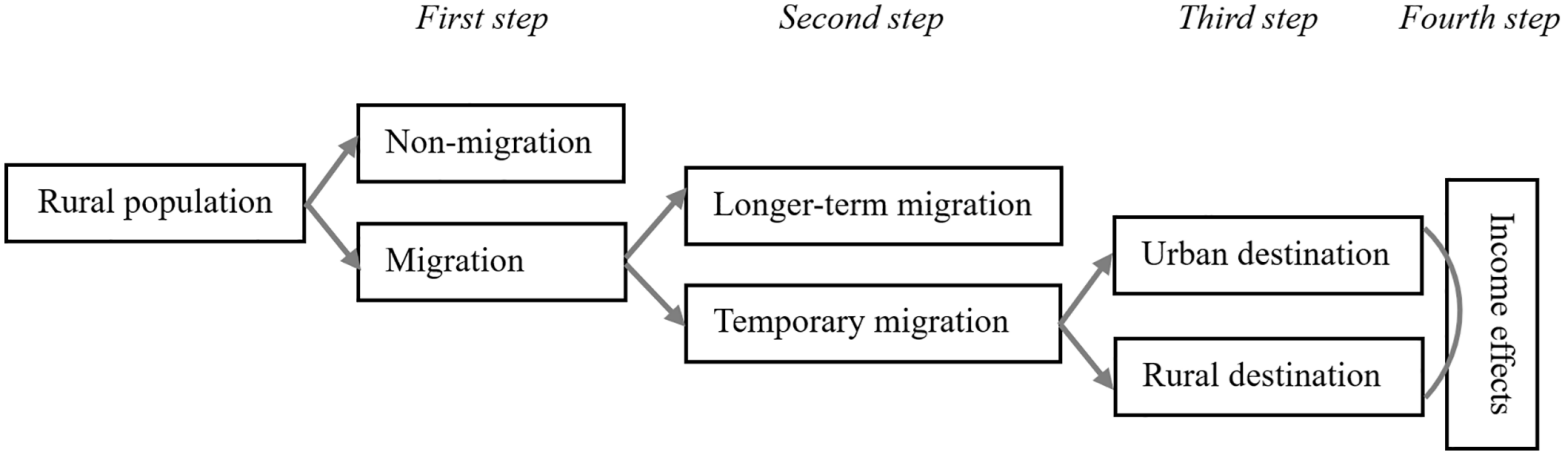
Conceptual framework.

Households’ participation in migration is self-selected. Similarly, the intra-household decision-making regarding a member’s migration is also not random [11,36]. Therefore, to understand temporary migrants’ destination choices (Objective 1) and the household-level income effects of different destination choices (Objective 2), it is crucial to estimate migration decisions across different steps (Fig 1), correcting for self-selection bias at each step.

Accordingly, we utilize a multi-step conditional regression analysis with subsamples, extending the Heckman selection model limited to two stages. In this approach, potential self-selection effects are estimated as the inverse Mills ratio (*imr*), following [37]. After estimating a binary outcome model, a probit model in our case, we predict the linear predictor for individual *i*’s participation (${xb}_{i}$). This predictor is then used to calculate the inverse Mills ratio for individual *i* (${imr}_{i}$), using equation (1) below, following [37]:


$$\begin{array}{c} {imr}_{i}=\;\frac{\phi ({xb}_{i})}{\text{1}-\;\Phi ({xb}_{i})} \end{array}$$ (1)

where, ϕ*(*${xb}_{i}$*)* and Φ*(*${xb}_{i}$*)* are the probability density function (PDF) and the cumulative distribution function (CDF) of the standard normal distribution evaluated at ${xb}_{i}$, respectively.

This ${imr}_{i}$ is then incorporated in the subsequent regression step to correct for potential self-selection bias. At the third step (Fig 1), we achieve our first objective, while the second one at the fourth step. Our multi-step conditional regression models are specified in the following.

#### Modeling destination choices during temporary migration.

For the first research objective, we utilize a three-step conditional probit selection model with subsamples in different equations. In the first migration step, equation (2), we utilize the entire individual dataset of 2,793 observations to model the participation of individual *i* in migration versus non-migration (*M*_*i*_). In the second step, equation (3), we utilize the subsample of 605 individual migrants to model their participation in temporary versus longer-term migration (*TM*_*i*_), incorporating their self-selection into migration (*imr1*_*i*_) that we calculate from equation (2) based on equation (1). In the third step, equation (4), we use only the subsample of 385 temporary migrants to model their choice of rural versus urban destinations (*R*_*i*_), incorporating their self-selection into temporary migration (*imr2*_*i*_), as calculated from equation (3). The three-step conditional probit equations are specified as below:


$$\begin{array}{c} M_{i}=\alpha \;(x_{ij},c_{jk},\;{ev}_{j})+u_{i} \end{array}$$ (2)

$$\begin{array}{c} {TM}_{i}=\beta \;(x_{ij},c_{jk},\;{imr\text{1}}_{i})+e_{i} \end{array}$$ (3)

$$\begin{array}{c} R_{i}=\delta \;(v_{i},c_{jk},\;{imr\text{2}}_{i})+\mu_{i} \end{array}$$ (4)

In equation (2), we account for the characteristics of individual *i* and household *j* ($x_{ij}$) relevant for individual *i*’s participation in migration, as conceptualized in the literature [9,10,38]. This vector includes migrant *i*’s individual characteristics such as age, education, gender, and primary occupation type, and household *j*’s characteristics such as its experience of seasonal employment fluctuations, farm labor or family obligations, and the size of migrant networks at the origin. In this equation, we also account for relevant other controls for household *j* and village *k* ($c_{jk}$). They include household size, wealth, access to alternative livelihoods, and proximity to nearby migration hubs. Additionally, they include some relevant village-level controls such as, whether the village is in a flood-prone area, and village-level fixed effects. For consistent estimates, this vector of $c_{jk}$ is controlled for in all subsequent equations.

Equation (3) models the individual migrant *i*’s selection into temporary versus longer-term migration (${TM}_{i}$) by accounting for similar vectors of $x_{ij}$ and $c_{jk}$, and the selection effects in migration- ${imr\text{1}}_{i}$, as calculated from equation (2). Since we use similar sets of explanatory variables in both equations (2) and (3), for a robust estimation of the selection effect- ${imr\text{1}}_{i}$, we utilize an exclusionary variable (${ev}_{j}$) in equation (2), as suggested by [37]. We use households’ experience of random economic shocks in their crops, livestock, and assets in the past 12 months as the exclusionary variable. These idiosyncratic economic shocks can sometimes restrict their capability of sending migrants [9,10]. However, these shocks are unlikely to affect households’ choice between the physical labor-based temporary and longer-term migration, if they have already decided about migration [9]. Another type of idiosyncratic shock includes the sudden death or severe accident of a working household member—family demographic shocks that may affect individual migrant’s choice between temporary and longer-term migration due to increased family obligations at the origin [29]. We separate these shocks and use only economic shocks as the exclusionary variable here. Table A2 in the Online Appendix confirms that the experience of random economic shocks differs significantly between migrant and non-migrants ($M_{i}$), but not between temporary and longer-term migrants (${TM}_{i}$), confirming our hypothesis.

Finally, equation (4) models the temporary migrants’ choice of rural versus urban destinations in their latest migration episode by correcting their self-selection into temporary migration (${imr\text{2}}_{i}$). In this equation, we incorporate *v*_*i*_ as the vector of relevant explanatory variables for individual migrant *i*’s choice of destination, informed by the relevant literature [9,29]. This vector includes migrant *i*’s individual characteristics- *I*_*i*_*,* negative perception of urban areas- *U*_*i*_*,* experience of the latest destination- *D*_*i*_*,* migrant networks- *N*_*i*_*,* and distance travelled in the latest migration episode- *Dist*_*i*_. These variables are described in Table 1. The parameters to be estimated in the respective equations are represented by α, *β*, and *ẟ*, and the error term by *u*_*i,*_ $e_{i}$*,* and $\mu_{i}$. Since equations (3) and (4) include distinct sets of explanatory variables, we do not introduce any additional exclusionary variable in equation (3), apart from the self-selection into migration (${imr\text{1}}_{i}$).

**Table 1:** Variables for analyzing destination choices during temporary migration.

Variables	Description	Expected sign in the model (Rural vs urban destination, $R_{i}$)
**Individual characteristics (*I***_*i*_)		
Education	Education in schooling years (1–14)	(-)
Agriculture labor sale	Engagement in agriculture labor sale at the origin (1/0)	(+)
**Urban negativity (*U***_***i***_ )*		
Prior negative perception of cities	Perception of ‘difficulty’ for living and earning in urban destinations before making the first migration (1/0).	(+)
**Experience of destination characteristics (*D***_*i*_ )*		
Income-to-cost ratio	Experience of the income-to-cost ratio at the latest destination (Likert scale of 1–10),	(+)
Physical comfort	Experience of physical comfort at the latest destination (Likert scale of 1–10),	(+)
**Migrant networks (*N***_*i*_)		
Rural-bound migrant kin	Have migrant kin or relatives migrating to rural destinations (1/0).	(+)
Migrant group size*	Size of the migrant group in the latest migration episode.	(+)
**Migration distance (*Dist***_*i*_)		
Travel distance	Physical distance (km) between the migrant’s origin and destination sub-districts.	(+/-)

* These data are subject to recall bias; however, this applies equally to both rural- and urban-bound temporary migrants, mitigating the likelihood of systematic bias.

Regarding individual characteristics (*I*_*i*_), studies find that individuals with higher education are more prone to longer-term migration, or at least to urban destinations during temporary migration [9,29], as education rarely brings extra benefits in rural destinations. Educated individuals often possess increased life-skills making them confident about better opportunities in cities. Conversely, individuals with lower or no education often lack life-skills, leading to a preference for simpler settings like in rural destinations. For better estimates about the association of individuals’ education with their destination choices, we also account for their skills (discussed in detail below) in this model.

Similarly, individuals engaged in agriculture labor at the origin may prefer on-farm jobs in other rural destinations due to socio-cultural familiarity [25]. We use individuals’ engagement in farm labor sales at the origin as a proxy for this preference (Table 1). However, any physical sensitivity to agricultural jobs—such as an inability to bend the waist for rice harvesting—may discourage sensitive individuals from choosing rural destinations, which we control for in the model. Moreover, we account for other relevant factors both at the individual (e.g., age) and household levels (e.g., household size, agricultural landholdings, family demographic shocks, and engagement in crop farming, livestock farming, business, safety-nets, and microcredit).

Regarding urban negativity (*U*_*i*_), individuals with lower education or lacking skills beyond agriculture often view urban destinations as a difficult place for earning and living [39]. This negative perception of cities may discourage aspiring migrants with lower education or skills from choosing urban destinations. Therefore, apart from education, we also account for migrants’ lack of life-skills beyond agriculture.

For individual migrants’ experiences at their latest migration destination (*D*_*i*_), we collect their subjective experience ratings on a 1–10 Likert scale, where 1 denotes ‘worst’ and 10 denotes ‘best’. For example, a migrant rating 10 for income-to-cost ratio characteristic means they could save most of their daily earnings at the latest migration destination. This often occurs in rural destinations in Bangladesh, where employers frequently offer free accommodation and meals for migrant laborers. Conversely, in urban destinations, around half of daily wages typically go toward accommodation and meal expenses [29], shifting their income-to-cost experience closer to ‘worst.’ Therefore, although wage opportunities are higher in urban areas, rural destinations may offer greater psychological satisfaction from saving ‘hard-earned’ income, influencing poor migrant laborers’ destination choices—a concept similar to ‘loss-aversion,’ which suggests that ‘losses’ have a greater influence on preferences than ‘gains’ [40].

Similarly, while jobs in both types of destinations can be physically demanding, agricultural tasks in rural destinations may offer comparatively better physical comforts to the migrants from rural origins. In contrast, urban destinations often provide longer-duration wage opportunities than rural ones [4]. This is particularly encouraging for temporary migrants from flood-prone villages, where lean periods are often prolonged due to weather extreme [5,9]. Therefore, we also account for migrants’ subjective experiences concerning wage opportunity duration at their latest migration destination, the geographic location of the village in flood-prone areas, and village fixed effects.

Regarding migrant networks (*N*_*i*_), in addition to the influence of migrant kin, the size of the migrant group from the origin may play a key role in choosing destinations [29]. A larger group size reduces rural poor’s risk-aversion toward migration and makes their migration pleasurable. Rural-bound migrants often travel in large groups, which is frequently required for employment in rural destinations. Employers in these areas tend to prefer hiring larger groups of migrant laborers to keep up with their crop calendar. Conversely, migration in larger groups may raise competition for jobs at rickshaw garages or construction sites in urban destinations. Group migration, therefore, could be more closely associated with rural-bound temporary migration, potentially encouraging risk-averse rural poor to prefer rural destinations. For this analysis, we use data on group size from migrants’ latest migration episode.

Regarding the implications of migration distance (*Dist*_*i*_) for destination choices, earlier studies have found that longer-distance permanent migrations move towards urban centers [11,26]. However, the relationship between distance and destination choices during temporary migration remains unclear, which we address here. We collect data on the destination sub-district for each migrant’s latest migration episode. The physical distance in kilometer (km) between the origin and destination sub-districts is then calculated using a geo-referencing system. Here, we mainly use the bus-road distance, as buses are the common transport mode across the country. Migration distance is treated as a proxy for migration costs. Moreover, we control for the proximity of individual migrants’ household to the nearest migration hub, often the closest sub-district.

Due to the relatively large number of explanatory variables included in the model, we test for multicollinearity by calculating the variance inflation factors for each equation. The results are shown in Table A3 in the Online Appendix. They do not indicate a high correlation among the explanatory variables and selection effects.

To check the robustness of our findings, we employ a system of simultaneous mixed-process equations using limited information maximum likelihood (LIML), following [41]. When multiple equations are mutually interdependent and deal with subsamples in different equations, as in our case, this analytical approach proves useful. For this analysis, we use the *cmp* command in Stata, incorporating equation (2)–(4) while excluding their respective *imr*s. We skip the likelihood-ratio test, use five random draws for the Geweke-Hajivassiliou-Keane (GHK) simulator, and apply the Newton-Raphson method for optimization.

Furthermore, migrant network (*N*_*i*_) and urban negativity (*U*_*i*_) variables may remain potentially endogenous in both models discussed above. To assess their sensitivity, we re-estimate the main model (Eq. 4) excluding these variables sequentially.

#### Modeling the income effects of different destination choices.

For our second research objective (fourth step, Fig 1), we utilize the same individual-level dataset to measure the income effects. We use the lean period income of household *j* (*Inc*_*j*_) as the outcome variable in this analysis, as temporary migration takes place mainly during the lean period [5,7,42]. The income effects of the individual migrant *i*’s choice of rural versus urban destination during temporary migration ($R_{i}$) is captured in equation (5) below:


$$\begin{array}{c} {Inc}_{j}=\theta \;(R_{i},\;z_{jk},\;{imr\text{3}}_{i})+\varepsilon_{i} \end{array}$$ (5)

Studies have shown that earning a lot of remittance is often not a priority for constrained poor temporary migrants [39]. Therefore, to better understand the income effects (*Inc*_*j*_) of destination choices during temporary migration, we use three indicators of income: i) total lean-period income of household *j* from all sources (*tot_inc*_*j*_), ii) income earned exclusively from migration remittances (*remit_inc*_*j*_), and iii) income from the origin’s labor market (*loc_inc*_*j*_ = *tot_inc*_*j*_*- remit_inc*_*j*_). Income amounts, categorized by seasons and sources, were collected in Bangladeshi Taka (BDT) currency. For this analysis, we apply a logarithmic transformation to the income data.

To obtain a more consistent estimate of the income effects, we control for certain relevant household and village characteristics for income ($z_{jk}$) in equation (5). These characteristics include the household head’s age, education, and gender, household size, and experience of seasonal employment fluctuation. They also include some village-level factors such as the location of the village in flood-prone areas and village fixed effects. The parameters are represented by *θ*, and the error term by $\varepsilon_{i}$. From equation (5), we report the coefficient (*θ*) for choosing rural versus urban destinations ($R_{i}$) as the estimated effects on the households’ lean period income (*Inc*_*j*_).

In equation (5), we also account for the migrant’s self-selection into destinations- *imr3*_*i*_, which is calculated from equation (4) based on equation (1). However, *imr3*_*i*_ appears insignificant in equation (5) for all indicators of income, as presented in Table A7 in the Online Appendix. This suggests that self-selection into destinations may not be a challenging issue when estimating the income effects of different destination choices. Nevertheless, we cannot entirely rule out the possibility of endogeneity in destination choice, particularly arising from unobserved heterogeneity. To address this challenge, we apply a control function approach, which is effective in correcting this type of endogeneity [43].

In this approach, we calculate the control function or residuals (*res*) from equation (2)–(4) and incorporate them into subsequent equations in place of *imr*s. While *imr* is useful to correct self-selection bias, residuals account for the endogeneity arising from unobserved factors, mentioned above, by capturing the part of participation that is not explained by the controlled variables in the respective equation. To calculate residuals, after estimating a regression, we predict the probability of participation for individual *i* ($\overset{*}{\underset{i}{p}}$). Next, we calculate the residual for individual *i*’s participation (*res*_*i*_) as the difference between the observed value of participation ($p_{i}$) and the predicted probability of participation ($\overset{*}{\underset{i}{p}}$), as outlined in equation (6) below:


$$\begin{array}{c} {res}_{i}=p_{i}-\;\overset{*}{\underset{i}{p}} \end{array}$$ (6)

In our multi-step control function analysis, we calculate *res1*_*i*_ from migration equation (2) based on equation (6) and incorporate it into temporary migration equation (3), replacing *imr1*_*i*_. Similarly, *res2*_*i*_ is calculated from equation (3) and used in destination choice equation (4). Finally, we calculate *res3*_*i*_ from this equation (4) and incorporate it into income effects equation (5). This analysis uses a similar exclusionary variable (*ev*_*j*_) design, as discussed earlier in the multi-step conditional probit selection model.

Using control function approach, *res3*_*i*_ in equation (5) appears significant for some income indicators, as shown in row (1) of Table 5 in the results section, indicating the presence of endogeneity. Additionally, *res1*_*i*_ and *res2*_*i*_ appear significant in the respective equations, as presented in Table A8 in the Online Appendix.

To check robustness of our results from the multi-step control function analysis, we employ a similar approach of simultaneous mixed-process equations with LIML, as discussed earlier. Additionally, we use a two-stage least square (2sls) analysis with an instrumental variable (IV) design. The general two-stage equations for this analysis are outlined in equation (7) and (8) below:


$$\begin{array}{c} \text{First stage}:\;\overset{*}{\underset{i}{R}}=\delta \;\left(v_{i},c_{jk},\;z_{jk},\;{IV}_{i},\;{imr\text{2}}_{i}\right)+\mu_{i} \end{array}$$ (7)

$$\begin{array}{c} {\text{Second stage}:\;Inc}_{j}=\theta \;(\overset{*}{\underset{i}{R}},\;v_{i},c_{jk},\;z_{jk})+\varepsilon_{i} \end{array}$$ (8)

where, $\overset{*}{\underset{i}{R}}$ represents migrant *i*’s instrumented choice of rural versus urban destination, and *IV*_*i*_ denotes the instruments. We use the presence of rural-bound temporary migrant kin or relatives (see Table 1 for details) as an instrument here. This instrument is expected to influence migrant *i*’s choice of rural over urban destinations (*R*_*i*_) through network effects, but not to directly affect household income through channels other than networks. The first-stage regression results, presented in Table A10 in the Online Appendix, confirm the relevance of the instrument, with an F-statistic of 76.47 indicating its strength.

## Empirical results and discussions

### Descriptive statistics

**Fig 2:**
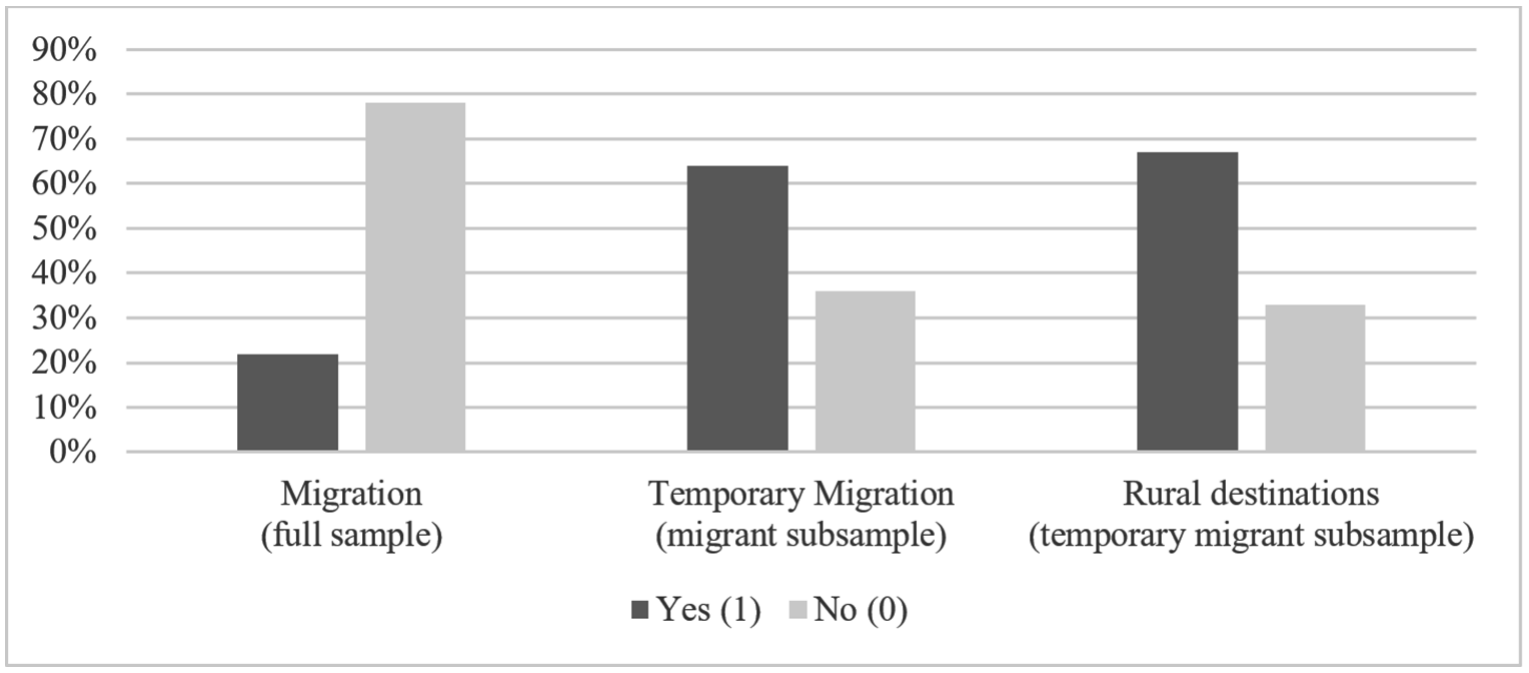
Migration and destination choice statistic.

Our data reveal that many migrations in northern rural Bangladesh are temporary and many of these temporary migrations follow rural destinations, as illustrated in Fig 2.

Among rural-bound temporary migrants in our sample, approximately 19% migrated to the Bogra district (~112 km from Rangpur city), 17% to the Tangail district (~218 km), 15% to the Comilla district (~402 km), and the rest to 26 other districts across the country. In contrast, Dhaka (~296 km from Rangpur), the capital city of Bangladesh with the largest urban agglomeration, appears to be the most attractive destination among urban-bound temporary migrants. About 62% of them chose Dhaka in their latest migration episode, while the rest were almost evenly distributed among 21 other cities/towns across the country.

Popular wage opportunities in rural destinations includes planting/harvesting rice paddy, working in other crop fields and brick kilns, among others. About 85% of our rural-bound temporary migrants were engaged in planting/harvesting rice during their latest migration episode. In contrast, about 48% of urban-bound migrants worked in masonry/construction sites, and around 39% in rickshaw-pulling (transportation) in cities.

The summary statistics of the key explanatory variables for destination choices during temporary migration are presented in Table 2 below. A test of mean differences between rural- and urban-bound temporary migrants generally supports our hypothesized associations between destination choices and indicators of individual characteristics (*I*_*i*_), urban negativity (*U*_*i*_), experience of destination characteristics (*D*_*i*_), and migrant networks (*N*_*i*_). Additionally, migration distance (*Dist*_*i*_) shows a significant negative association with the choice of rural destinations, broadly aligning with the existing literature.

**Table 2:** Summary statistics of the key explanatory variables for destination choices.

Variables	(1) All observations (n = 385)	(2) Rural-bound temporary migrants (n = 259)	(3) Urban-bound temporary migrants (n = 126)	(4) Mean difference (2–3)
**Individual characteristics (*I***_*i*_)				
Education	3.42 (3.74)	2.66 (3.18)	4.97 (4.30)	−2.31*** [0.39]
Agriculture labor sale	0.85 (0.36)	0.93 (0.25)	0.67 (0.47)	0.26*** [0.04]
**Urban negativity (*U***_***i***_ **)**				
Prior negative perception of cities	0.34 (0.48)	0.49 (0.50)	0.03 (0.18)	0.46*** [0.05]
**Experience of destination characteristics (D**_*i*_)				
Income-to-cost ratio	6.24 (3.14)	7.32 (2.93)	4.02 (2.26)	3.30*** [0.30]
Physical comfort	6.61 (3.24)	7.05 (3.23)	5.71 (3.09)	1.34*** [0.35]
**Migrant networks (*N***_*i*_)				
Rural-bound migrant kin	0.64 (0.48)	0.90 (0.30)	0.11 (0.32)	0.79*** [0.03]
Migrant group size	6.92 (5.04)	8.31 (5.06)	4.05 (3.59)	4.27*** [0.50]
**Migration distance (*Dist***_*i*_)				
Travel distance	274.82 (130.49)	251.58 (134.27)	322.60 (108.07)	−71.02*** [13.72]

Standard deviation in parentheses (); standard errors in square brackets []; *p < 0.10, **p < 0.05, ***p < 0.01.

Regarding the second research objective, we observe an insignificant mean difference in households’ total income during the lean period (*tot_inc*_*j*_) between rural- and urban-bound temporary migrant households (Table 3). However, the mean income from migration remittances (*remit_inc*_*j*_) and income from the local labor market (*loc_inc*_*j*_) differ significantly between these two groups. Households sending temporary migrants to urban areas appear to receive larger remittances than those sending to rural areas. Conversely, rural-bound temporary migrant households tend to have higher income from the origin’s local labor markets compared to those with urban-bound migrants.

**Table 3:** Mean household income for different destination choices.

Income variables (${Inc}_{j}$)	(1) All observations (n = 385)	(2) Rural-bound temporary migrants (n = 259)	(3) Urban-bound temporary migrants (n = 126)	(4) Mean difference (2–3)
Total income (*tot_inc*_*j*_)	4.00 (0.65)	3.98 (0.64)	4.03 (0.67)	−0.05 [0.07]
Remittance income (*remit_inc*_*j*_)	3.09 (0.92)	2.98 (0.86)	3.31 (1.00)	−0.34*** [0.10]
Local market income (*loc_inc*_*j*_)	2.95 (1.34)	3.11 (1.23)	2.61 (1.49)	0.51*** [0.14]

Standard deviation in parentheses (); standard errors in square brackets []; *p < 0.10, **p < 0.05, ***p < 0.01.

Note: Income in Bangladeshi Taka (BDT) and transformed to log; 1 USD = 122 BDT.

A plausible mechanism for these effects could be the duration of temporary migration. Approximately 80% of rural-bound temporary migrants in our sample migrated for a shorter duration of less than 30 days during their latest episode, possibly because wage opportunities are short-term and fragmented in rural destinations [17]. In contrast, around 57% of urban-bound temporary migrants stayed for longer than 30 days, suggesting that urban-bound temporary migrants tend to remain longer at destinations, enabling them to generate higher remittances—broadly consistent with the existing literature [4]. Rural-bound temporary migrants, in turn, may diversify risks by engaging in the origin’s labor market before undertaking shorter-duration migration. Table A4 in the Online Appendix shows that temporary migration episodes lasting less than 30 days are significantly associated with lower remittances and higher income from the local labor market, which are plausible.

Local market income options include both on-farm and off-farm opportunities. Table A5 in the Online Appendix presents that rural-bound temporary migrants earn significantly more from livestock farming and labor sales at the origin. These income effects are further elaborated in the regression results section.

### Regression results

Here, we present and discuss the regression results. First, we discuss individual migrants’ destination choices during temporary migration (*R*_*i*_). Next, we analyze the comparative income effects (*Inc*_*j*_) associated with these destination choices. These results should be interpreted as associations rather than causal relationships.

#### Destination choices during temporary migration.

As mentioned earlier, our three-step conditional probit selection model addresses three key questions: (i) why the rural people choose to migrate (*M*_*i*_, eq. 2), (ii) why they opt for temporary as opposed to longer-term migration (*TM*_*i*_*,* eq. 3), and (iii) why they prefer rural over urban destinations during temporary migration (*R*_*i*_, eq. 4). Results from equation (2) and (3) are presented in Table A6 in the Online Appendix. The choices of migration (eq. 2) and temporary versus longer-term migration (eq. 3) have already been studied in the literature, employing both qualitative and quantitative methodologies [9,13,16,29,38,44,45]. In brief, existing studies find that poor earnings at the origin and the presence of functional migration networks are important factors influencing the decision to migrate. Conversely, the presence of farm labor and family obligations discourages constrained households from migrating. However, when migration is necessary to diversify risks in a less-diversified economy, temporary migration is preferred, as it maximizes utility without exacerbating constraints. Our regression results align with existing literature despite drawing on different contexts and datasets.

The results from equation (4), which addresses our first research objective, are presented in column (1) of Table 4 below. Overall, most of our hypotheses on destination decision-making hold.

**Table 4:** Factors for destination choices during temporary migration.

Variables	(1) Multi-step conditional probit selection model with subsamples	(2) Simultaneous mixed process equations using LIML
	Rural vs urban destination choice (*R*_*i*_)	Rural vs urban destination choice (*R*_*i*_)
**Individual characteristics (*I***_*i*_)		
Education	−0.14*** [0.05]	−0.09** [0.04]
Agriculture labor sale	1.08** [0.44]	0.88*** [0.31]
**Relevant controls**		
Physical sensitivity to agriculture	−1.11** [0.47]	−1.07*** [0.40]
Age	0.00 [0.01]	−0.00 [0.01]
Household size	−0.21* [0.11]	−0.16* [0.09]
Agricultural landholdings	0.01 [0.02]	0.01 [0.02]
Crop farming	−0.69** [0.35]	−0.67** [0.32]
Livestock farming	0.76** [0.31]	0.63*** [0.24]
Family demographic shocks	−1.09** [0.50]	−0.79** [0.38]
Business	0.33 [0.35]	0.35 [0.34]
Social safety-nets	0.23 [0.31]	0.25 [0.26]
Having microcredit loans/memberships	−0.49* [0.28]	−0.44* [0.24]
**Urban negativity (*U***_***i***_)		
Prior negative perception of cities	1.10*** [0.37]	0.91*** [0.29]
**Relevant controls**		
Lack of skills beyond agriculture	0.77*** [0.27]	0.63*** [0.22]
**Experience of destination characteristics (*D***_*i*_)		
Income-to-cost ratio	0.36*** [0.06]	0.30*** [0.06]
Physical comfort	0.06 [0.05]	0.07* [0.04]
**Relevant controls**		
Daily wage opportunities	−0.21** [0.10]	−0.16** [0.08]
Flood vulnerability of the village	0.77 [0.73]	0.59 [0.49]
Village fixed effects	0.00 [0.00]	0.00 [0.00]
**Migrant networks (*N***_*i*_)		
Rural boundness of the closest migrant kin	3.02*** [0.44]	2.55*** [0.45]
Migrant group size	0.11*** [0.04]	0.08** [0.03]
**Migration distance (*Dist***_*i*_)		
Travel distance	−0.00*** [0.00]	−0.00*** [0.00]
**Relevant controls**		
Household distance to the nearby migration hub	−0.01 [0.01]	−0.01 [0.01]
imr2_*i*_	−0.72** [0.30]	
Constant	1.10 [1.66]	−0.27 [1.08]
Wald chi2	139.05	818.33

N=2,793; robust standard errors in square brackets- []; *p<0.10, **p<0.05, ***p<0.01.

Individual characteristics, such as low education and engagement in agricultural labor at the origin, are significantly associated with a preference for rural destinations during migration, as expected. Relevant household-level factors also play remarkable roles: livestock farming is associated with rural-bound migration, while crop farming has the opposite effect. This contrast likely reflects differences in labor requirements—livestock farming demands year-round family labor, discouraging longer duration migration such as to urban destinations, whereas crop-farming households can rely on hired local labor and still send migrants to urban areas for longer-duration wage opportunities [9,46]. We discuss this association further in the income effects section.

Negative perceptions of cities before the first migration, along with a lack of life-skills beyond agriculture, are significantly associated with choosing rural destinations, even in the latest migration episode. Moreover, a higher daily income-to-cost ratio (i.e., higher daily wages relative to expenses) appears to favor rural over urban destinations, although reverse causality is plausible. However, urban destinations are more strongly associated with wage opportunities, particularly for longer-duration employment. This suggests that rural destinations may be preferred for shorter and less costly migration episodes, whereas urban destinations may attract migrants seeking more sustained income-earning opportunities despite higher living costs. This also helps explain why many crop-farming households with relatively lower labor constraints are more inclined toward urban destinations during temporary migration.

Regarding migrant networks, the presence of kin or relatives migrating to rural destinations affects aspiring migrants’ destination preferences through network effects. Similarly, larger migrant group sizes are strongly associated with choosing rural over urban destinations, as expected, although endogeneity remains plausible in this context as well.

Also, distance is significantly associated with the destination choice. Our data reveal that rural-bound temporary migration is significantly more common over shorter distances, likely to minimize migration costs, which aligns with loss-aversion theory [40] and classical migration theories [11]. Shorter-distance migration also reduces the duration of migrants’ separation from their left-behind families. This is particularly encouraging for households that often face greater labor constraints for livestock farming and labor sales, along with increased family obligations due to a less flexible member structure (Table 4), consistent with the existing literature [9].

Selection effects (*imr*) are significant at every stage of the model, as shown in Table 4 above and Table A6 in the Online Appendix, justifying their correction. Results from the simultaneous mixed process equations, presented in column (2) of Table 4, are consistent with the main model, confirming its robustness. Moreover, the main results remain insensitive to potentially endogenous networks and perception variables, as demonstrated in Table A11 in the Online Appendix.

#### Income effects of the destination choice during temporary migration.

Results from the multi-step control function analysis, showing the income effects of destination choices during temporary migration (equation 5), are summarized in row (1) of Table 5. Results from equations (2)–(4) using this approach are presented in Table A8, and the full regression results for equation (5) are in Table A9 in the Online Appendix.

**Table 5:** Income effects of the destination choice during temporary migration.

Model	Variable	Total income *(tot_inc*_*j*_*)*	Remittance income *(remit_inc*_*j*_*)*	Local market income *(loc_inc*_*j*_*)*
(1) Multi-step control function analysis with subsamples	Rural over urban destination choice (*R*_*i*_)	−0.08 [0.08]	−0.39*** [0.12]	0.59*** [0.17]
	*res3*_*i*_	0.29* [0.16]	0.41 [0.40]	−0.30 [0.43]
	Constant	3.92*** [0.28]	2.85*** [0.36]	2.63*** [0.45]
	Controls (*z*_*i*_)	Yes	Yes	Yes
(2) Simultaneous mixed process equations using LIML	Rural over urban destination choice (*R*_*i*_)	−0.10 [0.09]	−0.46*** [0.13]	0.63*** [0.21]
	Constant	4.01*** [0.30]	3.17*** [0.34]	2.49*** [0.45]
	Controls (*z*_*i*_)	Yes	Yes	Yes
(3) Two-stage least square	Rural over urban destination choice (*R*_*i*_)	−0.45*** [0.17]	−0.52** [0.25]	0.31 [0.31]
	Constant	4.13*** [0.46]	1.80*** [0.62]	2.93*** [0.88]
	Controls (*z*_*i*_)	Yes	Yes	Yes

N = 2,793; Income in Bangladeshi Taka (BDT) and transformed to log; *res3*_*i*_- control function for individual migrant *i*’s participation in destinations; robust standard errors in square brackets- []; *p < 0.10, **p < 0.05, ***p < 0.01.

While temporary migration generally generates positive income gains for poorer households [9,47], we find that choosing rural over urban destinations is not significantly associated with higher total household income (*tot_inc*_*j*_). While rural destinations are often associated with better income-to-cost ratio than urban ones (Table 4), urban-bound migration generates higher remittances (*remit_inc*_*j*_). At the same time, we find that households choosing rural destinations earn significantly more from local labor market activities, particularly livestock farming and labor sales at the origin (Table A5). Similarly, equation (4) results show that engagement in these activities is positively associated with choosing rural over urban destinations (Table 4).These findings suggest that rural-bound temporary migration functions less as a remittance-maximization strategy and more as a complementary livelihood strategy that allows households to maintain stronger participation in origin-based economic activities while still benefiting from migration income, and more importantly, managing their labor constraints better through shorter-duration migration. By contrast, urban-bound migration appears to rely more heavily on remittances as a primary income source. In this sense, destination choice during temporary migration reflects different forms of household risk diversification and labor allocation, rather than a simple trade-off between higher and lower income.

One possible explanation is that some households face stronger farm labor or family constraints, limiting their ability to disengage from origin-based livelihoods for extended periods. For these households, rural destinations may offer a more compatible balance between migration and local economic participation, particularly given their more favorable income-to-cost conditions. Urban destinations, although potentially generating higher remittances, may require prolonged separation from origin-based labor activities and family obligations, with stronger dependence on migrant earnings alone.

These findings make a two-fold contribution to the literature: first, they clarify [29]’s ambiguous conclusion about the better income-to-cost ratio characteristic of rural destinations; and second, they shed light on the complex destination decision-making processes during temporary migration, which are often misunderstood from a monetary return’s perspective.

Results from the simultaneous mixed process equations, presented in row (2) of Table 5, are consistent with our main model. However, results from 2sls, presented in row (3) of Table 5, show significantly negative association between rural-bound temporary migration and total income, possibly because this group of households often belongs to the poorest segment of the community [4,5]. Similarly, although 2sls does not find a significant association between rural-bound migration and household income from the local labor market, it suggests a generally positive relationship, consistent with our main model.

## Conclusions and policy recommendations

Given that urban destinations often offer greater wage opportunities than their rural counterparts, understanding why many rural poor prefer rural destinations during their income-driven temporary migration remains crucial. We address this gap by first analyzing the motives behind this type of migration and then exploring its income effects. We use a multi-step conditional probit selection model with subsamples to analyze temporary migrants’ destination choices, correcting for their self-selection into migration and temporary migration. To address endogeneity in analyzing the income effects of different destination choices, we employ a multi-step control function approach with subsamples.

Under our first objective, we find that temporary migrants’ destination choices in northern Bangladesh are strongly associated with their individual characteristics, prior perceptions and subsequent experiences of the destination, migrant network influences, and household-level constraints, broadly consistent with existing studies [29,33].

Although rural destinations often offer a better income-to-cost ratio than urban ones, they are not necessarily superior to urban destinations in increasing total household income, as demonstrated under our second objective. By contrast, urban-bound temporary migration generates higher remittances, partly because this group of migrants tends to stay longer at destinations to maximize earnings. However, poor households facing farm labor and family constraints often prioritize shorter-duration migration during lean periods to optimally diversify risks by maintaining stronger engagement in the origin’s labor markets and spending more time with families. For such shorter-duration migration, rural destinations may provide a more compatible balance between migration income, migration costs, and household labor obligations. Together, these findings suggest that destination choices during temporary migration reflect broader household risk-diversification and labor allocation strategies rather than simple income maximization.

These findings are crucial for policies aimed at facilitating temporary migration as a risk diversification strategy for poor rural households. In particular, policies should support rural-bound temporary migration where prevalent, as many constrained, poor temporary migrants prefer this strategy after optimally exploiting local labor markets.

Furthermore, rural-to-rural migration can be crucial to address farm labor shortages in many destination rural areas, particularly in poor agrarian contexts like Bangladesh, where agricultural mechanization remains slow [48]. In recent years, the shortage of local agricultural laborers has been a major challenge in regions of Bangladesh that grow labor-intensive crops. This was evident during the COVID-19 pandemic when the harvest of the main crop—rice—was severely affected by a shortage of migrant laborers [49]. Facilitating rural-bound temporary migration between early- and late-harvesting rural areas or between labor-short and labor-surplus regions could help address this issue, as experienced in Bangladesh during the pandemic. Policy support could include providing wage information, reducing job search costs, and improving inter-district transportation networks to facilitate rural-to-rural migration.

Simultaneously, policies can expand local economies to improve the welfare of distress-driven migrants who rather prefer longer stays with their family at the origin. Promoting early harvesting rice varieties could reduce lean periods and migration duration. Also, crop diversification and off-farm employment generation could prove beneficial.

Future research could examine the effects of temporary migrant labor on crop production in destination rural areas. Another important avenue is to investigate how farm mechanization affects the livelihoods of agricultural labor–dependent rural households, who often rely on temporary migration as a key risk diversification strategy [9]. Finally, while this study focuses primarily on income effects, future work could also explore non-income returns to destination choices.

## Supporting information

S1 File Online appendix. (DOCX)

S2 File Data file. (ZIP)
