# Supplementary material for: Destination choices during internal temporary migration: Evidence from northern Bangladesh
Source: PLoS One. 2026 Jul 1;21(7):e0352665. doi: 10.1371/journal.pone.0352665 (PMC13322507; doi:10.1371/journal.pone.0352665)
Supplement: S1 File — (DOCX) [file pone.0352665.s001.docx]

**Online appendix**

**Destination Choices during Internal Temporary Migration: Evidence from Northern Bangladesh**

**Appendix A**

**Module 0: Consent [Mandatory]**

**0.1 Please read the full consent text to the respondent [Mandatory]**

As-salamu-alai-kum/Adab!

My name is *<enumerator name>,* I am a bachelor/master’s student from *<the institute name>.* I am assisting *<researcher name>* in collecting data for their study at the *<institute name>*. Your household has been RANDOMLY selected for this survey. Through this survey, we would like to know about *the kajer ovaber somoy* (lean periods) in your village and coping strategies of your household. Some of the questions are about your household’s income, asset, and household member’s details. If you do not want to answer these questions or any other questions, you can skip those questions or stop the survey at any time.

The survey may take around an hour to complete. Your participation in the survey is completely voluntary and it will not incur any costs to you except your time. By participating in this survey, you will not receive any incentives or benefits in return.

We are conducting this survey with over 600 households from 30 villages of *Dinajpur* and *Kurigram* Districts. All your data will be maintained with utmost confidentiality and used for research purpose only.

I shall start the survey only if you agree. If you want to know more about this study, or talk directly with the researcher, I can call *<the researcher>* now to connect you.

0.2 Do you have any question at this stage? **[Mandatory]** Yes No

0.3 Do you consent to participate in the survey? **[Mandatory]** Yes No

If **No**, end the survey here and go to the next randomly selected household.

**Table A1:** Sample distribution

| **District** | **Village** | **Sample size** |
| --- | --- | --- |
| Dinajpur | Ketra | 20 |
|  | Lotan | 29 |
|  | Dakkhin Madhabpur | 43 |
|  | Akhapur | 15 |
|  | Majhina | 24 |
|  | Maligram | 48 |
|  | Sindurhata | 23 |
|  | Laxmanpur | 22 |
|  | Banial | 24 |
|  | Nirmail | 28 |
|  | Daulgaon | 47 |
|  | Disbandi | 21 |
|  | Debipur | 27 |
|  | Dalaikota | 37 |
|  | Digalpara | 27 |
|  | Jagannathpur | 33 |
| Kurigram | Madhya Ganarkuthi | 34 |
|  | Kamat Angaria: Bashni Para | 41 |
|  | Kharkharia: Mandal Para | 38 |
|  | Madhya Kumarpur: Hudurdoba | 17 |
|  | Baraibari | 23 |
|  | Shalmara: Korani Para | 23 |
|  | Mollar Bhita | 31 |
|  | Barabhita: Dulabari | 25 |
|  | Shyampur: Kutibari | 20 |
|  | Taluk Asharu: Kuti | 23 |
|  | Dakshin Pranpati | 24 |
|  | Baldamara | 50 |
|  | Abdul Hadi | 35 |
|  | Dwarika | 26 |
| **Total** | **30 villages** | **878** |

**Table A2:** Mean of household experience of idiosyncratic economic shocks in the past year

| **Using individual-level dataset (n=2,793)** | | | | | | | |
| --- | --- | --- | --- | --- | --- | --- | --- |
| **Variable** | **(1)**  **All observations (n=2,793)** | **(2) Migrants**  **(n=605)** | **(3)**  **Non-migrants (n=2,188)** | **(4)**  **Mean difference (2-3)** | **(5) Temporary migrants**  **(n=385)** | **(6) Longer-term migrants**  **(n=220)** | **(7)**  **Mean difference (5-6)** |
| Experience of random economic shocks (${ev}_{j}$) | 0.35  (0.48) | 0.30  (0.46) | 0.36  (0.48) | -0.06***  [0.02] | 0.30  (0.46) | 0.31  (0.46) | -0.01  [0.04] |

Standard deviation in parentheses (); standard error in square brackets []; *p<0.10, **p<0.05, ***p<0.01

**Table A3:** Collinearity tests for variables to explain temporary migrant’s destination choices

| **Variables** | **Variance Inflation Factor (VIF)** | | |
| --- | --- | --- | --- |
|  | **Migration vs non-migration (*M_i_*)** | **Temporary vs longer-term migration (*TM_i_*)** | **Rural vs urban destination choice (*R_i_*)** |
| **Individual characteristics *(I_i_)*** |  |  |  |
| Education | 1.81 | 1.79 | 1.75 |
| Agricultural labor sale |  |  | 1.41 |
| **Relevant controls** |  |  |  |
| Physical sensitivity to agriculture |  |  | 1.25 |
| Age | 1.86 | 2.57 | 1.58 |
| Household size | 1.40 | 1.50 | 1.21 |
| Agricultural landholdings | 1.24 | 1.28 | 1.22 |
| Crop farming | 1.21 | 1.77 | 1.26 |
| Livestock farming | 1.16 | 1.54 | 1.17 |
| Family demographic shocks | 1.01 | 1.07 | 1.07 |
| Business | 1.12 | 1.47 | 1.09 |
| Social safety-nets | 1.09 | 1.22 | 1.14 |
| Microcredit memberships | 1.06 | 1.05 | 1.11 |
| **Urban negativity *(U_i_)*** |  |  |  |
| Prior negative perception of cities |  |  | 1.44 |
| **Relevant controls** |  |  |  |
| Lack of skills beyond agriculture |  |  | 1.42 |
| **Experience of destination characteristics *(D_i_)*** |  |  |  |
| Income-to-cost ratio |  |  | 1.38 |
| Physical comfort |  |  | 1.18 |
| **Relevant controls** |  |  |  |
| Daily wage opportunities |  |  | 1.09 |
| Flood vulnerability of the village | 1.16 | 1.20 | 1.19 |
| Village-level fixed effects | 1.30 | 1.65 | 1.34 |
| **Migrant networks *(N_i_)*** |  |  |  |
| Rural boundness of the closest migrant kin |  |  | 1.61 |
| Migrant group size |  |  | 1.38 |
| **Migration distance *(Dist_i_)*** |  |  |  |
| Travel distance (km) |  |  | 1.15 |
| **Relevant controls** |  |  |  |
| Household distance to the nearby migration hub | 1.12 | 1.14 | 1.16 |
| **Other controls (*X_i_*)** |  |  |  |
| Gender: Male | 1.55 |  |  |
| Occupation: Labor sales | 1.60 | 3.94 |  |
| Occupation: Farming | 1.46 | 2.09 |  |
| Seasonal employment fluctuation at the origin | 1.14 | 1.16 |  |
| Children | 1.18 | 1.31 |  |
| Elderly | 1.31 | 1.24 |  |
| Distrust in neighbors | 1.12 | 1.78 |  |
| Size of the migrant network | 1.12 | 2.82 |  |
| Random economic shocks (*ev_j_*) | 1.05 |  |  |
| *imr_i_* |  | 7.59 | 2.17 |
| Mean VIF | 1.28 | 1.96 | 1.32 |
| N | 2,793 | 605 | 385 |

**Table A4**: Mean household income for different temporary migration duration

| **Income variables (**${Inc}_{j}$**)** | **(1) All observations (n=385)** | **(2) <30 days duration in an episode**  **(n=258)** | **(3) >30 days duration in an episode**  **(n=127)** | **(4) Mean difference (2-3)** |
| --- | --- | --- | --- | --- |
| Total income (*tot_inc_j_*) | 4.00 (0.65) | 3.97 (0.65) | 4.06 (0.65) | -0.10 [0.07] |
| Remittance income (*remit_inc_j_*) | 3.09 (0.92) | 2.91 (0.90) | 3.45 (0.86) | -0.53*** [0.10] |
| Local market income (*loc_inc_j_*) | 2.95 (1.34) | 3.09 (1.28) | 2.65 (1.41) | 0.45*** [0.14] |

Standard deviation in parentheses (); standard errors in square brackets []; *p<0.10, **p<0.05, ***p<0.01

**Table A5**: Mean differences of income from different local market sources

| **Income sources** | **(1) All observations (n=385)** | **(2) Rural-bound temporary migrants**  **(n=259)** | **(3) Urban-bound temporary migrants (n=126)** | **(4) Mean difference (2-3)** |
| --- | --- | --- | --- | --- |
| Crop farming | 0.37 (1.07) | 0.42 (1.15) | 0.27 (0.90) | 0.15 [0.12] |
| Livestock farming | 0.37 (1.06) | 0.45 (1.14) | 0.20 (0.83) | 0.25** [0.11] |
| Labor sale | 2.14 (1.49) | 2.32 (1.42) | 1.76 (1.57) | 0.55*** [0.16] |
| Business | 0.46 (1.19) | 0.43 (1.16) | 0.54 (1.24) | -0.11 [0.13] |
| Monthly fixed/service | 0.27 (0.74) | 0.23 (0.67) | 0.35 (0.85) | -0.12 [0.08] |
| Seasonal safety-nets | 0.32 (0.69) | 0.33 (0.70) | 0.30 (0.67) | 0.03 [0.07] |
| Rents and assets | 0.03 (0.34) | 0.03 (0.38) | 0.02 (0.25) | 0.01 [0.04] |
| Others | 0.01 (0.19) | 0.01 (0.23) | 0.00 (0.00) | 0.01 [0.02] |

Standard deviation in parentheses (); standard errors in square brackets []; *p<0.10, **p<0.05, ***p<0.01

**Table A6**: Factors for migration and temporary migration by correcting self-selection (equation 2 and 3)

| **Variables** | **Migration vs non-migration (*M_i_*); eq. (2)** | **Temporary vs longer-term migration (*TM_i_*); eq. (3)** |
| --- | --- | --- |
| Age | -0.02*** [0.00] | 0.03*** [0.01] |
| Education | -0.01 [0.01] | -0.05*** [0.02] |
| Occupation: Farming | 0.31*** [0.11] | 1.11*** [0.28] |
| Occupation: Labor sales | 0.52*** [0.09] | 0.57** [0.25] |
| Seasonal employment fluctuation at the origin | 0.04 [0.07] | 0.70*** [0.14] |
| Children | 0.04 [0.07] | 0.35** [0.16] |
| Elderly | -0.07 [0.08] | 0.41** [0.18] |
| Distrust in neighbors | -0.84*** [0.10] | 1.78*** [0.39] |
| Crop farming | -0.33*** [0.08] | 0.53*** [0.18] |
| Livestock farming | -0.27*** [0.08] | 0.58*** [0.17] |
| Family demographic shocks | -0.32 [0.29] | -0.46 [0.63] |
| Size of the migrant network | 0.05*** [0.01] | 0.02 [0.02] |
| Household size | 0.00 [0.02] | -0.17*** [0.05] |
| Agricultural landholdings | -0.00 [0.00] | -0.01 [0.01] |
| Business | -0.35*** [0.09] | 0.47** [0.19] |
| Social safety-nets | -0.14* [0.08] | 0.01 [0.17] |
| Having microcredit loans/memberships | 0.04 [0.08] | 0.03 [0.16] |
| Household distance to the nearby migration hub | 0.00 [0.00] | -0.01* [0.00] |
| Flood vulnerability of the village | -0.19 [0.12] | -0.38* [0.21] |
| Village-level fixed effects | 0.00*** [0.00] | -0.00 [0.00] |
| Gender: Male | 2.00*** [0.12] |  |
| Random economic shocks (*ev_j_*) | -0.13* [0.07] |  |
| *imr_i_* |  | 1.66*** [0.55] |
| Constant | -3.80*** [0.32] | -2.46*** [0.66] |
| Wald chi2 | 551.25 | 240.54 |
| Observations | 2,793 | 605 |

Robust standard errors in square brackets []; *p<0.10, **p<0.05, ***p<0.01

**Table A7**: Income effects of destination choices by correcting self-selection bias (equation 5)

| **Model** | **Variable** | **Total income *(tot_inc_j_)*** | **Remittance income *(remit_inc_j_)*** | **Local market income *(loc_inc_j_)*** |
| --- | --- | --- | --- | --- |
| Multi-step conditional probit selection model with subsamples | Rural over urban destination choice (*R_i_*) | 0.06 [0.10] | -0.22 [0.18] | 0.45* [0.23] |
|  | *imr3_i_* | -0.10 [0.08] | -0.11 [0.14] | 0.09 [0.18] |
|  | Constant | 3.89*** [0.28] | 2.79*** [0.36] | 2.67*** [0.45] |
|  | Controls (*z_i_*) | Yes | Yes | Yes |

N= 2,793; Standard errors in square brackets []; *p<0.10, **p<0.05, ***p<0.01

**Table A8**: Factors for migration, temporary migration, and destination choices employing multi-step control function approach (equation 2, 3, and 4)

| **Variables** | **Migration vs non-migration (*M_i_*); eq. (2)** | **Temporary vs longer-term migration (*TM_i_*); eq. (3)** | **Rural vs urban destination choice (*R_i_*); eq. (4)** |
| --- | --- | --- | --- |
| **Individual characteristics *(I_i_)*** |  |  |  |
| Education | -0.01 [0.01] | -0.05** [0.02] | -0.15*** [0.06] |
| Agricultural labor sale |  |  | 1.27*** [0.46] |
| **Relevant controls** |  |  |  |
| Physical sensitivity to agriculture |  |  | -1.16** [0.44] |
| Age | -0.02*** [0.00] | 0.05*** [0.01] | 0.01 [0.01] |
| Household size | 0.00 [0.02] | -0.18*** [0.05] | -0.26** [0.11] |
| Agricultural landholdings | -0.00 [0.00] | -0.01 [0.02] | 0.02 [0.02] |
| Crop farming | -0.33*** [0.08] | 0.79*** [0.26] | -0.80** [0.36] |
| Livestock farming | -0.27*** [0.08] | 0.80*** [0.22] | 0.76** [0.32] |
| Family demographic shocks | -0.32 [0.29] | -0.26 [0.71] | -1.02** [0.49] |
| Business | -0.35*** [0.09] | 0.76*** [0.27] | 0.33 [0.34] |
| Social safety-nets | -0.14* [0.08] | 0.10 [0.19] | 0.22 [0.32] |
| Having microcredit loans/memberships | 0.04 [0.08] | 0.00 [0.16] | -0.54* [0.30] |
| **Urban negativity *(U_i_)*** |  |  |  |
| Prior negative perception of cities |  |  | 1.15*** [0.36] |
| **Relevant controls** |  |  |  |
| Lack of skills beyond agriculture |  |  | 0.69** [0.27] |
| **Experience of destination characteristics *(D_i_)*** |  |  |  |
| Income-to-cost ratio |  |  | 0.38*** [0.07] |
| Physical comfort |  |  | 0.07 [0.05] |
| **Relevant controls** |  |  |  |
| Daily wage opportunities |  |  | -0.27*** [0.11] |
| Flood vulnerability of the village | -0.19 [0.12] | -0.28 [0.23] | 0.69 [0.72] |
| Village-level fixed effects | 0.00*** [0.00] | -0.00* [0.00] | 0.00 [0.00] |
| **Migrant networks *(N_i_)*** |  |  |  |
| Rural boundness of the closest migrant kin |  |  | 3.15*** [0.48] |
| Migrant group size |  |  | 0.11*** [0.04] |
| **Migration distance *(Dist_i_)*** |  |  |  |
| Travel distance (km) |  |  | -0.00*** [0.00] |
| **Relevant controls** |  |  |  |
| Household distance to the nearby migration hub | 0.00 [0.00] | -0.01** [0.00] | -0.01 [0.01] |
| **Other controls (*X_i_*)** |  |  |  |
| Gender: Male | 2.00*** [0.12] |  |  |
| Occupation: Labor sale | 0.52*** [0.09] | 0.14 [0.36] |  |
| Occupation: Farming | 0.31*** [0.11] | 0.82** [0.33] |  |
| Seasonal employment fluctuation at the origin | 0.04 [0.07] | 0.67*** [0.14] |  |
| Children | 0.04 [0.07] | 0.33** [0.16] |  |
| Elderly | -0.07 [0.08] | 0.51*** [0.19] |  |
| Distrust in neighbors | -0.84*** [0.10] | 2.54*** [0.57] |  |
| Size of the migrant network | 0.05*** [0.01] | -0.01 [0.04] |  |
| Random economic shocks (*ev_j_*) | -0.13* [0.07] |  |  |
| *res_i_* |  | -5.32*** [1.71] | 2.57*** [0.93] |
| Constant | -3.80*** [0.32] | 1.45 [0.97] | -0.01 [1.43] |
| Wald chi2 | 551.25 | 255.59 | 125.42 |
| Observations | 2,793 | 605 | 385 |

Standard errors in square brackets []; *p<0.10, **p<0.05, ***p<0.01

**Table A9:** Full regression results from equation (5) using multi-step control function approach

| **Variable** | **Total income *(tot_inc_j_)*** | **Remittance income *(remit_inc_j_)*** | **Local market income *(loc_inc_j_)*** |
| --- | --- | --- | --- |
| Rural over urban destination choice (*R_i_*) | -0.08 [0.08] | -0.39*** [0.12] | 0.59*** [0.17] |
| Age | -0.00 [0.00] | -0.00 [0.00] | 0.00 [0.01] |
| Education | 0.01 [0.01] | -0.00 [0.01] | 0.00 [0.02] |
| Gender: Male | 0.26* [0.15] | 0.10 [0.22] | -0.16 [0.24] |
| Household size | 0.04 [0.03] | 0.03 [0.04] | 0.12*** [0.04] |
| Seasonal employment fluctuation at the origin | -0.13* [0.07] | 0.18* [0.10] | -0.62*** [0.13] |
| Flood vulnerability of the village | 0.09 [0.10] | 0.17 [0.18] | 0.06 [0.18] |
| Village-level fixed effects | -0.00 [0.00] | 0.00* [0.00] | -0.00 [0.00] |
| *res3_i_* | 0.29* [0.16] | 0.41 [0.40] | -0.30 [0.43] |
| Constant | 3.92*** [0.28] | 2.85*** [0.36] | 2.63*** [0.45] |

N= 2,793; Standard errors in square brackets []; *p<0.10, **p<0.05, ***p<0.01

**Table A10**: First-stage regression results summary from 2sls

| **Variables** | **Choice of rural versus urban destination (*R_i_*)** |
| --- | --- |
| Instrument: Rural-bound migrant kin (1/0) | 0.54*** [0.05] |
| Constant | 0.60*** [0.16] |
| F-statistics | 76.47 |
| Controls | Yes |

N= 2,793; Standard errors in square brackets []; *p<0.10, **p<0.05, ***p<0.01

**Table A11:** Sensitivity analysis for destination choices (equation 4)

| **Variables** | **(1) Main model: Multi-step conditional probit selection model with subsamples** | **(2) Excluding migrant networks (*N_i_*)** | **(3) Excluding migrant networks (*N****_i_***) and urban negativity (*U****_i_***)** |
| --- | --- | --- | --- |
|  | Rural vs urban destination choice (*R_i_*) | Rural vs urban destination choice (*R_i_*) | Rural vs urban destination choice (*R_i_*) |
| **Individual characteristics (*I****_i_***)** |  |  |  |
| Education | -0.14*** [0.05] | -0.08** [0.03] | -0.09*** [0.03] |
| Agriculture labor sale | 1.08** [0.44] | 0.65** [0.29] | 0.78*** [0.27] |
| **Relevant controls** |  |  |  |
| Physical sensitivity to agriculture | -1.11** [0.47] | -1.81*** [0.43] | -1.88*** [0.38] |
| Age | 0.00 [0.01] | -0.00 [0.01] | -0.00 [0.01] |
| Household size | -0.21* [0.11] | -0.11 [0.07] | -0.08 [0.07] |
| Agricultural landholdings | 0.01 [0.02] | -0.02 [0.01] | -0.00 [0.02] |
| Crop farming | -0.69** [0.35] | -0.36 [0.24] | -0.50** [0.22] |
| Livestock farming | 0.76** [0.31] | 0.27 [0.21] | 0.37* [0.20] |
| Family demographic shocks | -1.09** [0.50] | -0.35 [0.60] | 0.37 [0.58] |
| Business | 0.33 [0.35] | 0.05 [0.27] | 0.00 [0.27] |
| Social safety-nets | 0.23 [0.31] | -0.02 [0.24] | -0.05 [0.22] |
| Having microcredit loans/memberships | -0.49* [0.28] | -0.36 [0.26] | -0.37* [0.22] |
| **Urban negativity (*U_i_*)** |  |  |  |
| Prior negative perception of cities | 1.10*** [0.37] | 1.36*** [0.34] | Excluded |
| **Relevant controls** |  |  |  |
| Lack of skills beyond agriculture | 0.77*** [0.27] | 0.92*** [0.28] | Excluded |
| **Experience of destination characteristics (*D****_i_***)** | |  |  |
| Income-to-cost ratio | 0.36*** [0.06] | 0.25*** [0.03] | 0.29*** [0.03] |
| Physical comfort | 0.06 [0.05] | 0.09*** [0.03] | 0.08** [0.03] |
| **Relevant controls** |  |  |  |
| Daily wage opportunities | -0.21** [0.10] | -0.27*** [0.10] | -0.26*** [0.09] |
| Flood vulnerability of the village | 0.77 [0.73] | 0.99*** [0.36] | 0.81** [0.34] |
| Village fixed effects | 0.00 [0.00] | -0.00 [0.00] | -0.00 [0.00] |
| **Migrant networks (*N****_i_***)** |  |  |  |
| Rural boundness of the closest migrant kin | 3.02*** [0.44] | Excluded | Excluded |
| Migrant group size | 0.11*** [0.04] | Excluded | Excluded |
| **Migration distance (*Dist****_i_***)** |  |  |  |
| Travel distance | -0.00*** [0.00] | -0.00*** [0.00] | -0.00*** [0.00] |
| **Relevant controls** |  |  |  |
| HH distance to the nearby migration hub | -0.01 [0.01] | -0.00 [0.01] | -0.00 [0.01] |
| imr2*_i_* | -0.72** [0.30] | -0.08 [0.16] | 0.13 [0.17] |
| Constant | 1.10 [1.66] | 2.93** [1.33] | 2.53** [1.19] |
| Wald chi2 | 139.05 | 146.73 | 154.04 |

N= 2,793; Standard errors in square brackets []; *p<0.10, **p<0.05, ***p<0.01.
