# Supplementary material for: Destination choices during internal temporary migration: Evidence from northern Bangladesh
Source: PLoS One. 2026 Jul 1;21(7):e0352665. doi: 10.1371/journal.pone.0352665 (PMC13322507; doi:10.1371/journal.pone.0352665)
Supplement: Attachment — Submitted filename: PLOS ONE Reviewer Comments.docx [file pone.0352665.s003.docx]

### ****PLOS ONE Reviewer Comments****

**Manuscript ID**: PONE-D-25-24890
**Title**: Destination choices during internal temporary migration: Evidence from northern Bangladesh

**Overall Evaluation**

This manuscript focuses on rural-to-rural temporary migration among rural populations in northern Bangladesh, a topic of significant practical relevance and certain academic novelty. The authors employ stratified sampling data and a multi-step conditional choice model, combined with a control function approach, to examine farmers' destination choices and the associated impacts on household income. The methodological design is reasonable, the data are substantial, and the quantitative models are generally sound. This study provides useful supplementary insights into understanding non-conventional migration patterns.

However, the overall theoretical framing and analytical dimensions of the manuscript remain relatively narrow. The authors rely heavily on economic rationality and social network perspectives, while neglecting institutional, environmental, and emotional factors. In addition, the policy recommendations fail to address deeper structural issues. Overall, I recommend substantial revision and improvement in the areas of theoretical framework, variable design, and result interpretation.

**Major Comments**

1.Overemphasis on Income and Neglect of Emotional Factors in Rural-to-Rural Migration

The manuscript overlooks emotional adaptation and cultural identity mechanisms in explaining migration decisions. For Bangladeshi rural migrants, moving to other rural areas is not only an economic decision but also reflects cultural familiarity and emotional security. Compared to unfamiliar urban environments, rural destinations offer continuity in language, religion, lifestyle, and social interaction patterns, thus providing a sense of belonging and emotional ease. The choice of rural destinations partially reflects farmers' avoidance of cultural heterogeneity and psychological insecurity associated with cities.

I recommend:

Incorporating emotional adaptation and cultural identity as analytical dimensions in the theoretical or discussion sections;

Citing relevant literature on migration and belonging, as well as emotional geographies of migration.

2.Neglect of Land Privatization and Land Tenure Insecurity

The manuscript fails to consider the structural impacts of land privatization and tenure insecurity on migration decisions. Since the 1980s, land market privatization in Bangladesh has led to:Land fragmentation; Increasing concentration of land ownership; Expansion of informal tenancy markets;Exclusion of marginal and landless farmers from productive land.

As a result, large numbers of rural poor households are compelled to engage in short-term agricultural labor migration to survive. Migration is not merely a seasonal income strategy, but also a structural outcome of land exclusion and market-driven land reforms.

I recommend:

Including land tenure and land market exclusion as structural factors in the analysis;

Reassessing migration formation mechanisms from the perspective of land privatization and land loss;

Referring to relevant studies on land tenure insecurity and agrarian transformation in South Asia.

3.Overly Simplified Classification of Destination Types

The current definition of “rural destinations” is unclear and overly broad. In practice, some destinations categorized as rural may include small towns or peri-urban spaces, reflecting distinct migration patterns.

I recommend:

Clarifying how “rural” is defined in the survey;

Refining the typology of destinations to avoid conflating heterogeneous migration pathways.

4.Overly Narrow Measurement of Migration Returns

Migration outcomes are measured solely in terms of household income and remittances. However, for land-constrained households, livelihood diversification and risk management are equally important motivations. I recommend:

Expanding the discussion of non-income-based returns, such as risk mitigation and livelihood security;

Clarifying that income maximization may not be the sole objective of all migration decisions.

5.Overly Narrow Policy Recommendations

The current policy recommendations focus primarily on supporting rural-to-rural migration. However, the structural drivers of forced migration, such as land exclusion and agricultural risks, are not addressed.

I recommend:

Proposing policies that mitigate agricultural seasonality and land tenure insecurity;

Supporting diversification of rural employment and strengthening land rights protections;

Developing small-town labor markets as alternative employment spaces;

Framing migration policy within a broader risk management and livelihood improvement strategy.

**Minor Comments**

Clarify whether land loss, environmental degradation, or pollution were considered in the survey instrument.

Specify whether indicators like “income-to-cost ratio” and “physical comfort” were based on subjective perceptions or objective measures.

Ensure consistency in describing the relationship between migration distance and migration costs.

**Recommendation**

Major Revision

After incorporating land tenure systems, environmental factors, emotional and cultural dimensions, expanding the theoretical framework, refining destination typologies, and revising the policy recommendations, this manuscript has potential for publication. I recommend major revision and resubmission.
