# Supplementary material for: Destination choices during internal temporary migration: Evidence from northern Bangladesh
Source: PLoS One. 2026 Jul 1;21(7):e0352665. doi: 10.1371/journal.pone.0352665 (PMC13322507; doi:10.1371/journal.pone.0352665)
Supplement: Attachment — Submitted filename: Reviewer_responses.docx [file pone.0352665.s004.docx]

### ****PLOS ONE Reviewer Comments****

**Manuscript ID**: PONE-D-25-24890
**Title**: Destination choices during internal temporary migration: Evidence from northern Bangladesh

**Overall Evaluation**

This manuscript focuses on rural-to-rural temporary migration among rural populations in northern Bangladesh, a topic of significant practical relevance and certain academic novelty. The authors employ stratified sampling data and a multi-step conditional choice model, combined with a control function approach, to examine farmers' destination choices and the associated impacts on household income. The methodological design is reasonable, the data are substantial, and the quantitative models are generally sound. This study provides useful supplementary insights into understanding non-conventional migration patterns.

However, the overall theoretical framing and analytical dimensions of the manuscript remain relatively narrow. The authors rely heavily on economic rationality and social network perspectives, while neglecting institutional, environmental, and emotional factors. In addition, the policy recommendations fail to address deeper structural issues. Overall, I recommend substantial revision and improvement in the areas of theoretical framework, variable design, and result interpretation.

**Major Comments**

***Comment 1. Overemphasis on Income and Neglect of Emotional Factors in Rural-to-Rural Migration***

The manuscript overlooks emotional adaptation and cultural identity mechanisms in explaining migration decisions. For Bangladeshi rural migrants, moving to other rural areas is not only an economic decision but also reflects cultural familiarity and emotional security. Compared to unfamiliar urban environments, rural destinations offer continuity in language, religion, lifestyle, and social interaction patterns, thus providing a sense of belonging and emotional ease. The choice of rural destinations partially reflects farmers' avoidance of cultural heterogeneity and psychological insecurity associated with cities.

I recommend:

Incorporating emotional adaptation and cultural identity as analytical dimensions in the theoretical or discussion sections;

Citing relevant literature on migration and belonging, as well as emotional geographies of migration.

**Responses:** In Bangladesh, Bengali is the most commonly spoken language, and more than 90% of population is Muslim in the major destination places. Therefore, we did not expect any significant variations on these indicators, hence did not control for them.

For cultural familiarity, and emotional security, we use proxies in the analyses. With a positive hypothesis about cultural familiarity and emotional security in rural destinations, we add migrant’s education, occupation in agriculture in the model (please refer to Table 1, Individual Characteristics (*I_i_*)). On p.9, we write, *“Educated individuals often possess increased life-skills making them confident about better opportunities in cities. Conversely, individuals with lower or no education often lack life-skills, leading to a preference for known and simpler settings like in rural destinations.”*

Later, on p.9, we also write, *“Similarly, individuals engaged in agriculture labor at the origin may prefer on-farm jobs in other rural destinations due to socio-cultural familiarity. We use individuals’ engagement in farm labor sales at the origin as a proxy for this preference (see Table 1). However, any physical sensitivity to agricultural jobs—such as an inability to bend the waist for rice harvesting—may discourage sensitive individuals from choosing rural destinations, which we control for in the model. Moreover, we account for other relevant factors both at the individual (e.g., age) and household levels (e.g., household size, agricultural landholdings, family demographic shocks, and engagement in crop farming, livestock farming, business, safety-nets, and microcredit).”*

Furthermore, household demographic factors/obligations, e.g., presence of children and elderly members requiring special care, distrust in neighbors for taking care of the left-behind family during migration, and smaller size of households triggering demographic constraints are also included in the model to control for migrant’s emotional security during migration. In the results section on p.19, we write: *“… As we have seen from equation (3) (Table A5 in the Online Appendix), farm labor and family obligations are critical factors in choosing temporary over longer-term migration, which is also consistent with the existing literature [9, 39]. Some of these households may have greater constraints that limit their migration duration. For these households, earning lot of remittances is often not a priority as mentioned earlier. They rather prefer longer stays with their families by optimally diversifying their risks at the origin before migrating.”*

Also, we have cited the most recent papers (to our knowledge) on destination choices during migration, as our objectives are in this direction.

***Comment 2. Neglect of Land Privatization and Land Tenure Insecurity***

The manuscript fails to consider the structural impacts of land privatization and tenure insecurity on migration decisions. Since the 1980s, land market privatization in Bangladesh has led to:Land fragmentation; Increasing concentration of land ownership; Expansion of informal tenancy markets; Exclusion of marginal and landless farmers from productive land.

As a result, large numbers of rural poor households are compelled to engage in short-term agricultural labor migration to survive. Migration is not merely a seasonal income strategy, but also a structural outcome of land exclusion and market-driven land reforms.

I recommend:

Including land tenure and land market exclusion as structural factors in the analysis;

Reassessing migration formation mechanisms from the perspective of land privatization and land loss;

Referring to relevant studies on land tenure insecurity and agrarian transformation in South Asia.

**Responses**: We agree that land ownership affects rural poor’s migration decision, particularly for choosing migration as opposed to non-migration. Similar results are also found in a recent study [Rana et al., 2025].

In our study, we have also controlled for households’ land ownership (captured by the variable “agricultural landholdings”) in all three equations: migration vs. non-migration, temporary vs. longer-term migration, and rural vs. urban destination selections. As shown in Table 4 in the results section (p. 17), land ownership does not significantly affect their choice of destinations. While we agree that agricultural land ownership could affect households’ migration decision, we could not derive any conceptual links between land ownership and destination choice during temporary migration. Accordingly, as our focus is on the latter, we do not elaborate on this variable in the discussion of this paper.

***Comment 3. Overly Simplified Classification of Destination Types***

The current definition of “rural destinations” is unclear and overly broad. In practice, some destinations categorized as rural may include small towns or peri-urban spaces, reflecting distinct migration patterns.

I recommend:

Clarifying how “rural” is defined in the survey;

Refining the typology of destinations to avoid conflating heterogeneous migration pathways.

**Responses:** We apologize for this oversight. In the revised version, and on p.6, now we define rural and urban destinations as follows:

*“Following the definition provided by the Bangladesh Bureau of Statistics [49], we classify urban destinations as areas located within the jurisdictions of City Corporations, Municipalities, Sub-district Headquarters (not declared as Municipalities), Cantonment areas, and Growth Centres enlisted by the Local Government Division. All other areas are classified as rural destinations. Additionally, we collect information on the type of work undertaken by migrants at their destinations, which we use to further differentiate between rural and urban destinations when migrants are unable to identify the destination type.”*

***Comment 4. Overly Narrow Measurement of Migration Returns***

Migration outcomes are measured solely in terms of household income and remittances. However, for land-constrained households, livelihood diversification and risk management are equally important motivations. I recommend:

Expanding the discussion of non-income-based returns, such as risk mitigation and livelihood security;

Clarifying that income maximization may not be the sole objective of all migration decisions.

**Responses**: This paper examines income effects only in relation to destination choice during temporary migration; a broader discussion of migration returns lies outside its scope. However, this might be addressed in future research. We write on p.22, “*…..Finally, while this study focuses primarily on income effects, future work could also explore non-income-related returns to destination choices.”*

For temporary migration, income is often the primary—if not the sole—purpose, as indicated in the literature (Coffey et al., 2014, Khandker and Mahmud, 2012,  Mishra, 2016, Rana & Qaim, 2024; Tiwari et al., 2022, Wang et al., 2021). Therefore, in this paper, we focus on this trait of temporary migration while interpreting income as a livelihood diversification strategy.

In this context, we find that rural-bound temporary migration is indeed an important risk diversification strategy for constrained, poor rural households. We write on p. 19-20 the following:

*“…. As we have seen from equation (3) (Table A5 in the Online Appendix), farm labor and family obligations are critical factors in choosing temporary over longer-term migration, which is also consistent with the existing literature [9, 39]. Some of these households may have greater constraints that limit their migration duration. For these households, earning lot of remittances is often not a priority as mentioned earlier. They rather prefer longer stays with their families by optimally diversifying their risks at the origin before migrating.*

*We have also demonstrated in equation (4) (Table 4) that households’ engagement in livestock farming and selling labor at the origin is positively and significantly associated with their choice of rural over urban destinations. Similarly, rural-bound temporary migrants earn significantly more from these two sources (Table A4 in the Online Appendix). This explains the significantly higher income from the local labor market (loc_inc_j_) for rural-bound temporary migrants, as presented in Table 5.*

*Some of these income options, particularly livestock farming, create significant labor constraints at the origin, prompting shorter-duration temporary migration, mentioned earlier. For such short-duration migration, rural destinations with a more favorable income-to-cost ratio allow migrants to maximize utility through gaining a greater satisfaction from their limited migration time and income. This includes the satisfaction to save their “hard-earned” income [29], balance household labor needs, and reduce time away from home. Conversely, urban destinations may offer higher total income but require longer stays to offset higher living costs, which can lower overall utility for migrants, particularly from households with farm labor or family constraints. In other words, rural-bound temporary migration enables more efficient trade-offs, supporting longer stays with the family and risks diversification at the origin, while urban-bound migration often relies solely on remittances.”*

***Comment 5. Overly Narrow Policy Recommendations***

The current policy recommendations focus primarily on supporting rural-to-rural migration. However, the structural drivers of forced migration, such as land exclusion and agricultural risks, are not addressed.

I recommend:

Proposing policies that mitigate agricultural seasonality and land tenure insecurity;

Supporting diversification of rural employment and strengthening land rights protections;

Developing small-town labor markets as alternative employment spaces;

Framing migration policy within a broader risk management and livelihood improvement strategy.

**Responses:** We agree on our overly narrow policy recommendations. In the revised version, on p. 22, we write aligning with our main objectives:

*“…….. Policy support could include providing wage information, reducing job search costs, and improving inter-district transportation networks.*

*Moreover, to support distress-driven temporary migrants, promoting early harvesting rice varieties could reduce lean periods. Expanding local economies to support rural livelihoods during these times is also essential.”*

**----------------------------------------------**

**Minor Comments**

**Comment 1)** Clarify whether land loss, environmental degradation, or pollution were considered in the survey instrument.

**Responses**: We controlled “per capita agricultural landholdings” in all models. However, we did not capture land loss in survey instruments.

Pollution was considered as a potential factor for “difficulty for living in cities”, thus incorporated in “Urban Negativity” variable (see Table 1 on p.9).

“Flood vulnerability of villages” is controlled in the model to proxy for environmental degradation.

**Comment 2)** Specify whether indicators like “income-to-cost ratio” and “physical comfort” were based on subjective perceptions or objective measures.

**Response**: These were subjective ratings in 1-10 Likert scale as explained on p. 10. We have also specified the “subjective” nature of these data in the revised version.

**Comment 3)** Ensure consistency in describing the relationship between migration distance and migration costs.

**Response:** In the revised version, on p.11, we specify: “Migration distance is treated as a proxy for migration costs.”

------------------------------------------------------------------------

**Reviewers’ comments copied from the email body:**

***Review Comments to the Author***

***Reviewer #1:*** I have read the article “Destination choices during internal temporary migration: Evidence from northern Bangladesh” with great interest.
Amid growing attention to rural poverty and seasonal hunger, the study is especially important for understanding non-urban destination preferences among poor migrants—an under-researched but relevant topic in migration studies. By focusing on temporary migration in northern Bangladesh, the article challenges prevailing assumptions that urban destinations always offer better economic outcomes. The research addresses empirical gaps by analyzing the utility maximization of rural-bound migrants, drawing implications for policy design targeting rural development and labor mobility.
***Specific comments:*** The methodology employed is suitable for addressing self-selection bias and potential endogeneity, although the model complexity risks over-specification. The choice of instruments and exclusion restrictions is mostly justified (e.g., random economic shocks as an exclusionary variable), yet some relationships (e.g., urban perception and network effects) remain potentially endogenous and could benefit from sensitivity analysis.

**Responses**: We agree. In the revised version, in Methodology section on p. 11, we write: *“….. Furthermore, migrant network (N_i_) and urban negativity (U_i_) variables may remain potentially endogenous in both models discussed above. To assess their sensitivity, we re-estimate the main model (Eq. 4) excluding these variables sequentially.”*

Later in the results section, on p. 18, we interpret the results: “*… the main results remain insensitive to potentially endogenous networks and perception variables, as demonstrated in Table A10 in the Online Appendix.”*

Also, we remain cautious in interpreting the results, emphasizing on association rather than causality. At the beginning of the results section, on p. 16, we write: *“These results should be interpreted as associations rather than causal relationships.”*

***Specific Comments:*** The results indicate that while rural destinations offer a better income-to-cost ratio, they do not maximize household income compared to urban migration. This reveals that destination choices are shaped not merely by wages but also by constraints like family obligations, duration flexibility, and network effects. The authors appropriately reference classical (e.g., Todaro, Lewis) and contemporary (e.g., de Haas) migration theories but could engage more with capability/aspiration frameworks and gender-sensitive perspectives, especially given the absence of female temporary migrants. The implications for labor policy and regional planning could be more deeply explored. These points are important to take into consideration if the authors choose to revise: potential recall bias in self-reported data; weak theorization of utility maximization; minimal gender analysis; over-reliance on perception data without triangulation; and limited exploration of long-term livelihood outcomes.
Some relevant literature seem to have been missed.

**Responses:**

***Gender-sensitive perspectives:*** Since no women participated in temporary migration in our dataset, we did not explain on this, as we focus on the destination choice for temporary migration. However, following your kind feedback, in the revised version, on p.6, we write the following:
*“Our dataset shows no participation of women in temporary migration. This likely reflects both the social stigma surrounding this type of migration and the growing availability of garment industry employment, which offers longer-term opportunities or women migrants with relatively better working conditions and social dignity [29, 49]. Among our longer-term migrants, only 9% are women, all of whom are employed in the garment sector.”*

***Perception data and recall bias:***

In the revised version, on p. 9, we identify variables that are subject to potential recall bias and write the following as a note to the variable description Table 1:

*“These data are subject to recall bias; however, this applies equally to both rural- and urban-bound temporary migrants, mitigating the likelihood of systematic bias.”*

Additionally, perception data may be subject to endogeneity. In the revised version, we check the sensitivity of our main results to these variables. Results, presented in Table A10 in the online appendix, confirm the robustness of our results.

***Theorization of utility maximization:*** In the revised version, on p. 20 in the results section, we have explained utility maximization for rural vs. urban-bound temporary migration as the following:

*“For such short-duration migration, rural destinations with a more favorable income-to-cost ratio allow migrants to maximize utility through gaining a greater satisfaction from their limited migration time and income. This includes the satisfaction to save their “hard-earned” income [29], balance household labor needs, and reduce time away from home. Conversely, urban destinations may offer higher total income but require longer stays to offset higher living costs, which can lower overall utility for migrants, particularly from households with farm labor or family constraints. In other words, rural-bound temporary migration enables more efficient trade-offs, supporting longer stays with the family and risks diversification at the origin, while urban-bound migration often relies solely on remittances.”*

***-----------------------------------***

***Reviewer #2:*** This manuscript focuses on rural-to-rural temporary migration among rural populations in northern Bangladesh, a topic of significant practical relevance and certain academic novelty. The authors employ stratified sampling data and a multi-step conditional choice model, combined with a control function approach, to examine farmers' destination choices and the associated impacts on household income. The methodological design is reasonable, the data are substantial, and the quantitative models are generally sound. This study provides useful supplementary insights into understanding non-conventional migration patterns.
However, the overall theoretical framing and analytical dimensions of the manuscript remain relatively narrow. The authors rely heavily on economic rationality and social network perspectives, while neglecting institutional, environmental, and emotional factors. In addition, the policy recommendations fail to address deeper structural issues. Overall, I recommend substantial revision and improvement in the areas of theoretical framework, variable design, and result interpretation.

**Responses:** Thank you for this useful feedback. We have addressed these in the “Major Comments” section above.

-----------------------------------------------------------------------------------

**Editor’s comment from the email body:**

1. You may seek permission from the original copyright holder of Figure A1 to publish the content specifically under the CC BY 4.0 license.

**Response:** The map is freely available from the Local Govt Engineering Department (LGED), Bangladesh. However, we also feel that this map does not add much value to the paper. Therefore, we removed it in the revised version.
