# Supplementary material for: Destination choices during internal temporary migration: Evidence from northern Bangladesh
Source: PLoS One. 2026 Jul 1;21(7):e0352665. doi: 10.1371/journal.pone.0352665 (PMC13322507; doi:10.1371/journal.pone.0352665)
Supplement: Attachment — Submitted filename: Reviewer feedback.docx [file pone.0352665.s005.docx]

**PONE-D-25-24890R1**

“Destination Choices during Internal Temporary Migration: Evidence from Northern Bangladesh”

**Reviewers' comments:**

***Review Comments to the Author***

Reviewer #3: (No Response)

Reviewer #4: I would like to thank the author(s) for their effort in preparing this manuscript. This is a commendable effort by the authors; however, there are some areas that require further refinement before the manuscript is ready for publication. My overall comments on the manuscript are as follows:

**Comment # 1**: In the Introduction, the research gap needs to be more clearly elaborated, along with an explanation of how the article addresses this gap. The research question is currently missing and should be explicitly stated. Additionally, the article's overall structure should be clearly outlined.

**Response:** Thank you for all your feedback. We outline research gaps and our contribution to the literature in row # 41-55 (page 3-4) as the following:

*“**Several studies have examined destination choices for internal migration, but mainly focusing on permanent or longer-term migration types, which differs in nature from temporary migration [24-28]. To our knowledge, only one study [29] examines destination choices for temporary migration, employing an exploratory qualitative methodology with a relatively small sample size. This study highlights the importance of migrants’ individual characteristics, their networks, and perceptions about destinations in shaping destination choices. Notably, this study finds greater income gain perception for rural relative to urban destination choices, questioning the common assumption that urban destinations offer greater gains.*

*Yet perceived gains may not necessarily correspond to actual income effects. Destination choices may instead reflect differences in migration costs, remittance patterns, and households’ need to balance migration with origin-based livelihood activities, aspects that remain unclear in the existing literature. By examining both the determinants and income effects of temporary rural versus urban migration, we investigate whether destination choices are primarily driven by realized economic returns or by broader household risk-diversification strategies.*

*We employ a quantitative methodology to achieve this.……”*

We outline our research question in the row # 38-40 (page 3) as below:

*“…..**This raises a fundamental question as to why do many temporary migrants prefer rural over urban destinations, a topic poorly understood in the existing literature, which we address in this paper.”*

The article’s overall structure is outlined in row # 58-61 (page 4) as below:

*“**We organize this article as follows: Section 2 presents sampling, data, conceptual framework, and models used to achieve our research objectives. The empirical results are presented and discussed in Section 3. Finally, we conclude the article and outline its policy implications in Section 4.”*

**Comment # 2**: The article should include a dedicated section on the conceptual framework. This section should provide a brief review of relevant literature and explain how household-level migration decisions are determined.

**Response:** For the brevity, we have merged conceptual framework with model specifications. Clarified in the text, row # 162 (page 8). Also, we have included the conceptual framework graph (Figure 2, page 8) and explained it while specifying our models. Existing literature are also discussed in this section while building the model equations and explaining variables (page 9-14).

**Comment # 3**: In the Materials and Methods section, a map should be included to illustrate the geographical context of the study area. The rationale for selecting these specific locations should also be clearly justified. Additionally, a table should be provided to present the sample distribution by district, upazila, and union. The sampling process—particularly the selection of households—needs to be explained in detail. A separate table should also be included to present the socio-demographic profile of the sample households.

**Response:** Study area map is included in Figure 1 (page 5). We use a multi-stage sampling procedure to select study areas and collect household data. Rationale for selecting study areas and household sampling procedures is explained in “2.1 Sampling procedure” section, row # 63-104 (page 4-6). Sample distribution is presented in Table A1 in the Online Appendix.

We add a paragraph outlining relevant socio-demographic information of the sampled households in the section “2.3 Socio-demographic and migration data” (row # 129-135, page 7).

**Comment # 4**: A Discussion section should be included to interpret the results and explain how they relate to the broader literature. This section should highlight both the similarities with existing studies and the differences from them, along with possible reasons for these variations.

**Response:** For the brevity, we have merged discussions with descriptive and regression results in Section “3. Empirical results and discussions”.

We have highlighted similarities of our findings with the existing literature where necessary, such as Footnote # 6 (page 18), row # 478, 526, and 541.

In the destination choice equation (4), we do not find notable variations from the existing studies. Our income effect equation (5) is novel, and we use its findings and those from equation (4) to clarify destination decision-making process, which is our contribution to the literature. We have clarified our contributions in the Results section as below:

“*These findings make a two-fold contribution to the literature: first, they clarify [29]’s ambiguous conclusion about the better income-to-cost ratio characteristic of rural destinations; and second, they shed light on the complex destination decision-making processes during temporary migration, which are often misunderstood from a monetary return’s perspective.”* (row # 518-521, page 22).

Then in the conclusions, we summarize our contributions as the following:

*“Together, these findings suggest that destination choices during temporary migration reflect broader household risk-diversification and labor allocation strategies rather than simple income maximization.”* (row # 551-553, page 23).

We also highlight the similar in the abstract, row # 14-19.

**Comment # 5**: Citations should be avoided in the Conclusion section. It should begin by briefly stating what the article has addressed. The section should then clearly summarize the study's key findings and explain how these results align with the article’s objectives. Furthermore, the conclusion should highlight how the findings contribute to policy development—not only in Bangladesh but also in broader global contexts. Finally, it should outline potential directions for future research.

**Response:** Citations have been minimized in the Conclusion section. In the first paragraph, row # 530 to 537 (page 22), we briefly state what our study addresses. In the next two paragraphs, row # 538-553 (page 22-23), we discuss our findings aligning with the research objectives.

Policy implications of the paper are explained in the next three paragraphs, row # 554-572 (page 23). Policy recommendations are made for poor agrarian contexts, exemplifying with Bangladesh, where these recommendations are more contextually relevant.

Future research directions are outlined in the last paragraph, row # 573-578 (page 24).
