# Supplementary material for: Destination choices during internal temporary migration: Evidence from northern Bangladesh
Source: PLoS One. 2026 Jul 1;21(7):e0352665. doi: 10.1371/journal.pone.0352665 (PMC13322507; doi:10.1371/journal.pone.0352665)
Supplement: Peer review history

# Peer review: Decision Letter 0

**DOI:** 10.1371/journal.pone.0352665.r001

**Authors:** Tianlong You

22 Jul 2025

PONE-D-25-24890

Destination choices during internal temporary migration: Evidence from northern Bangladesh

PLOS ONE

Dear Dr. Rana,

Thank you for submitting your manuscript to PLOS ONE. After careful consideration, we feel that it has merit but does not fully meet PLOS ONE’s publication criteria as it currently stands. Therefore, we invite you to submit a revised version of the manuscript that addresses the points raised during the review process.

Please submit your revised manuscript by Sep 05 2025 11:59PM. If you will need more time than this to complete your revisions, please reply to this message or contact the journal office at plosone@plos.org. When you're ready to submit your revision, log on to https://www.editorialmanager.com/pone/ and select the 'Submissions Needing Revision' folder to locate your manuscript file.

Please include the following items when submitting your revised manuscript:

- A rebuttal letter that responds to each point raised by the academic editor and reviewer(s). You should upload this letter as a separate file labeled 'Response to Reviewers'.
- A marked-up copy of your manuscript that highlights changes made to the original version. You should upload this as a separate file labeled 'Revised Manuscript with Track Changes'.
- An unmarked version of your revised paper without tracked changes. You should upload this as a separate file labeled 'Manuscript'.

If you would like to make changes to your financial disclosure, please include your updated statement in your cover letter. Guidelines for resubmitting your figure files are available below the reviewer comments at the end of this letter.

If applicable, we recommend that you deposit your laboratory protocols in protocols.io to enhance the reproducibility of your results. Protocols.io assigns your protocol its own identifier (DOI) so that it can be cited independently in the future. For instructions see: https://journals.plos.org/plosone/s/submission-guidelines#loc-laboratory-protocols. Additionally, PLOS ONE offers an option for publishing peer-reviewed Lab Protocol articles, which describe protocols hosted on protocols.io. Read more information on sharing protocols at https://plos.org/protocols?utm_medium=editorial-email&utm_source=authorletters&utm_campaign=protocols.

We look forward to receiving your revised manuscript.

Kind regards,

Tianlong You, Ph.D.

Academic Editor

PLOS ONE

Journal Requirements:

When submitting your revision, we need you to address these additional requirements.

1.Please ensure that your manuscript meets PLOS ONE's style requirements, including those for file naming. The PLOS ONE style templates can be found at

https://journals.plos.org/plosone/s/file?id=wjVg/PLOSOne_formatting_sample_main_body.pdf and

https://journals.plos.org/plosone/s/file?id=ba62/PLOSOne_formatting_sample_title_authors_affiliations.pdf

2. Thank you for stating the following financial disclosure:

“Rana, MS received funding from the German Academic Exchange Service (DAAD) and the Foundation Fiat Panis for this study.”

Please state what role the funders took in the study.  If the funders had no role, please state: "The funders had no role in study design, data collection and analysis, decision to publish, or preparation of the manuscript."

If this statement is not correct you must amend it as needed.

Please include this amended Role of Funder statement in your cover letter; we will change the online submission form on your behalf.

3. In the online submission form, you indicated that “The data will be available upon request.”

All PLOS journals now require all data underlying the findings described in their manuscript to be freely available to other researchers, either 1. In a public repository, 2. Within the manuscript itself, or 3. Uploaded as supplementary information.

This policy applies to all data except where public deposition would breach compliance with the protocol approved by your research ethics board. If your data cannot be made publicly available for ethical or legal reasons (e.g., public availability would compromise patient privacy), please explain your reasons on resubmission and your exemption request will be escalated for approval.

4. We note that Figure A1 in your submission contain map/satellite images which may be copyrighted. All PLOS content is published under the Creative Commons Attribution License (CC BY 4.0), which means that the manuscript, images, and Supporting Information files will be freely available online, and any third party is permitted to access, download, copy, distribute, and use these materials in any way, even commercially, with proper attribution. For these reasons, we cannot publish previously copyrighted maps or satellite images created using proprietary data, such as Google software (Google Maps, Street View, and Earth). For more information, see our copyright guidelines: http://journals.plos.org/plosone/s/licenses-and-copyright.

We require you to either (1) present written permission from the copyright holder to publish these figures specifically under the CC BY 4.0 license, or (2) remove the figures from your submission:

1. You may seek permission from the original copyright holder of Figure A1 to publish the content specifically under the CC BY 4.0 license.

We recommend that you contact the original copyright holder with the Content Permission Form (http://journals.plos.org/plosone/s/file?id=7c09/content-permission-form.pdf) and the following text:

“I request permission for the open-access journal PLOS ONE to publish XXX under the Creative Commons Attribution License (CCAL) CC BY 4.0 (http://creativecommons.org/licenses/by/4.0/). Please be aware that this license allows unrestricted use and distribution, even commercially, by third parties. Please reply and provide explicit written permission to publish XXX under a CC BY license and complete the attached form.”

Please upload the completed Content Permission Form or other proof of granted permissions as an "Other" file with your submission.

In the figure caption of the copyrighted figure, please include the following text: “Reprinted from [ref] under a CC BY license, with permission from [name of publisher], original copyright [original copyright year].”

2. If you are unable to obtain permission from the original copyright holder to publish these figures under the CC BY 4.0 license or if the copyright holder’s requirements are incompatible with the CC BY 4.0 license, please either i) remove the figure or ii) supply a replacement figure that complies with the CC BY 4.0 license. Please check copyright information on all replacement figures and update the figure caption with source information. If applicable, please specify in the figure caption text when a figure is similar but not identical to the original image and is therefore for illustrative purposes only.

The following resources for replacing copyrighted map figures may be helpful:

USGS National Map Viewer (public domain): http://viewer.nationalmap.gov/viewer/

The Gateway to Astronaut Photography of Earth (public domain): http://eol.jsc.nasa.gov/sseop/clickmap/

Maps at the CIA (public domain): https://www.cia.gov/library/publications/the-world-factbook/index.html and https://www.cia.gov/library/publications/cia-maps-publications/index.html

NASA Earth Observatory (public domain): http://earthobservatory.nasa.gov/

Landsat: http://landsat.visibleearth.nasa.gov/

USGS EROS (Earth Resources Observatory and Science (EROS) Center) (public domain): http://eros.usgs.gov/#

Natural Earth (public domain): http://www.naturalearthdata.com/

5. If the reviewer comments include a recommendation to cite specific previously published works, please review and evaluate these publications to determine whether they are relevant and should be cited. There is no requirement to cite these works unless the editor has indicated otherwise.

[Note: HTML markup is below. Please do not edit.]

Reviewers' comments:

Reviewer's Responses to Questions

**Comments to the Author**

1. Is the manuscript technically sound, and do the data support the conclusions?

The manuscript must describe a technically sound piece of scientific research with data that supports the conclusions. Experiments must have been conducted rigorously, with appropriate controls, replication, and sample sizes. The conclusions must be drawn appropriately based on the data presented.

Reviewer #1: Partly

Reviewer #2: Yes

**********

2. Has the statistical analysis been performed appropriately and rigorously?

Reviewer #1: Yes

Reviewer #2: Yes

**********

3. Have the authors made all data underlying the findings in their manuscript fully available?

The PLOS Data policy requires authors to make all data underlying the findings described in their manuscript fully available without restriction, with rare exception (please refer to the Data Availability Statement in the manuscript PDF file). The data should be provided as part of the manuscript or its supporting information, or deposited to a public repository. For example, in addition to summary statistics, the data points behind means, medians and variance measures should be available. If there are restrictions on publicly sharing data—e.g. participant privacy or use of data from a third party—those must be specified.

Reviewer #1: Yes

Reviewer #2: Yes

**********

4. Is the manuscript presented in an intelligible fashion and written in standard English?

PLOS ONE does not copyedit accepted manuscripts, so the language in submitted articles must be clear, correct, and unambiguous. Any typographical or grammatical errors should be corrected at revision, so please note any specific errors here.

Reviewer #1: Yes

Reviewer #2: Yes

**********

5. Review Comments to the Author

Please use the space provided to explain your answers to the questions above. You may also include additional comments for the author, including concerns about dual publication, research ethics, or publication ethics. (Please upload your review as an attachment if it exceeds 20,000 characters)

Reviewer #1: I have read the article “Destination choices during internal temporary migration: Evidence from northern Bangladesh” with great interest.

Amid growing attention to rural poverty and seasonal hunger, the study is especially important for understanding non-urban destination preferences among poor migrants—an under-researched but relevant topic in migration studies. By focusing on temporary migration in northern Bangladesh, the article challenges prevailing assumptions that urban destinations always offer better economic outcomes. The research addresses empirical gaps by analyzing the utility maximization of rural-bound migrants, drawing implications for policy design targeting rural development and labor mobility.

The methodology employed is suitable for addressing self-selection bias and potential endogeneity, although the model complexity risks over-specification. The choice of instruments and exclusion restrictions is mostly justified (e.g., random economic shocks as an exclusionary variable), yet some relationships (e.g., urban perception and network effects) remain potentially endogenous and could benefit from sensitivity analysis.

The results indicate that while rural destinations offer a better income-to-cost ratio, they do not maximize household income compared to urban migration. This reveals that destination choices are shaped not merely by wages but also by constraints like family obligations, duration flexibility, and network effects. The authors appropriately reference classical (e.g., Todaro, Lewis) and contemporary (e.g., de Haas) migration theories but could engage more with capability/aspiration frameworks and gender-sensitive perspectives, especially given the absence of female temporary migrants. The implications for labor policy and regional planning could be more deeply explored. These points are important to take into consideration if the authors choose to revise: potential recall bias in self-reported data; weak theorization of utility maximization; minimal gender analysis; over-reliance on perception data without triangulation; and limited exploration of long-term livelihood outcomes.

Some relevant literaure seem to have been missed.

Reviewer #2: This manuscript focuses on rural-to-rural temporary migration among rural populations in northern Bangladesh, a topic of significant practical relevance and certain academic novelty. The authors employ stratified sampling data and a multi-step conditional choice model, combined with a control function approach, to examine farmers' destination choices and the associated impacts on household income. The methodological design is reasonable, the data are substantial, and the quantitative models are generally sound. This study provides useful supplementary insights into understanding non-conventional migration patterns.

However, the overall theoretical framing and analytical dimensions of the manuscript remain relatively narrow. The authors rely heavily on economic rationality and social network perspectives, while neglecting institutional, environmental, and emotional factors. In addition, the policy recommendations fail to address deeper structural issues. Overall, I recommend substantial revision and improvement in the areas of theoretical framework, variable design, and result interpretation.

**********

6. PLOS authors have the option to publish the peer review history of their article (what does this mean?). If published, this will include your full peer review and any attached files.

If you choose “no”, your identity will remain anonymous but your review may still be made public.

**Do you want your identity to be public for this peer review?**  For information about this choice, including consent withdrawal, please see our Privacy Policy.

Reviewer #1: **Yes:**  AKM AHSAN ULLAH

Reviewer #2: **Yes:**  Fangliang Zhang

**********

[NOTE: If reviewer comments were submitted as an attachment file, they will be attached to this email and accessible via the submission site. Please log into your account, locate the manuscript record, and check for the action link "View Attachments". If this link does not appear, there are no attachment files.]

While revising your submission, please upload your figure files to the Preflight Analysis and Conversion Engine (PACE) digital diagnostic tool, https://pacev2.apexcovantage.com/. PACE helps ensure that figures meet PLOS requirements. To use PACE, you must first register as a user. Registration is free. Then, login and navigate to the UPLOAD tab, where you will find detailed instructions on how to use the tool. If you encounter any issues or have any questions when using PACE, please email PLOS at figures@plos.org. Please note that Supporting Information files do not need this step.

Attachment Submitted filename: PLOS ONE Reviewer Comments.docx

# Author comment: Author response to Decision Letter 1

**DOI:** 10.1371/journal.pone.0352665.r002

6 Oct 2025

PLOS ONE Reviewer Comments

Manuscript ID: PONE-D-25-24890

Title: Destination choices during internal temporary migration: Evidence from northern Bangladesh

Overall Evaluation

This manuscript focuses on rural-to-rural temporary migration among rural populations in northern Bangladesh, a topic of significant practical relevance and certain academic novelty. The authors employ stratified sampling data and a multi-step conditional choice model, combined with a control function approach, to examine farmers' destination choices and the associated impacts on household income. The methodological design is reasonable, the data are substantial, and the quantitative models are generally sound. This study provides useful supplementary insights into understanding non-conventional migration patterns.

However, the overall theoretical framing and analytical dimensions of the manuscript remain relatively narrow. The authors rely heavily on economic rationality and social network perspectives, while neglecting institutional, environmental, and emotional factors. In addition, the policy recommendations fail to address deeper structural issues. Overall, I recommend substantial revision and improvement in the areas of theoretical framework, variable design, and result interpretation.

Major Comments

Comment 1. Overemphasis on Income and Neglect of Emotional Factors in Rural-to-Rural Migration

The manuscript overlooks emotional adaptation and cultural identity mechanisms in explaining migration decisions. For Bangladeshi rural migrants, moving to other rural areas is not only an economic decision but also reflects cultural familiarity and emotional security. Compared to unfamiliar urban environments, rural destinations offer continuity in language, religion, lifestyle, and social interaction patterns, thus providing a sense of belonging and emotional ease. The choice of rural destinations partially reflects farmers' avoidance of cultural heterogeneity and psychological insecurity associated with cities.

I recommend:

Incorporating emotional adaptation and cultural identity as analytical dimensions in the theoretical or discussion sections;

Citing relevant literature on migration and belonging, as well as emotional geographies of migration.

Responses: In Bangladesh, Bengali is the most commonly spoken language, and more than 90% of population is Muslim in the major destination places. Therefore, we did not expect any significant variations on these indicators, hence did not control for them.

For cultural familiarity, and emotional security, we use proxies in the analyses. With a positive hypothesis about cultural familiarity and emotional security in rural destinations, we add migrant’s education, occupation in agriculture in the model (please refer to Table 1, Individual Characteristics (Ii)). On p.9, we write, “Educated individuals often possess increased life-skills making them confident about better opportunities in cities. Conversely, individuals with lower or no education often lack life-skills, leading to a preference for known and simpler settings like in rural destinations.”

Later, on p.9, we also write, “Similarly, individuals engaged in agriculture labor at the origin may prefer on-farm jobs in other rural destinations due to socio-cultural familiarity. We use individuals’ engagement in farm labor sales at the origin as a proxy for this preference (see Table 1). However, any physical sensitivity to agricultural jobs—such as an inability to bend the waist for rice harvesting—may discourage sensitive individuals from choosing rural destinations, which we control for in the model. Moreover, we account for other relevant factors both at the individual (e.g., age) and household levels (e.g., household size, agricultural landholdings, family demographic shocks, and engagement in crop farming, livestock farming, business, safety-nets, and microcredit).”

Furthermore, household demographic factors/obligations, e.g., presence of children and elderly members requiring special care, distrust in neighbors for taking care of the left-behind family during migration, and smaller size of households triggering demographic constraints are also included in the model to control for migrant’s emotional security during migration. In the results section on p.19, we write: “… As we have seen from equation (3) (Table A5 in the Online Appendix), farm labor and family obligations are critical factors in choosing temporary over longer-term migration, which is also consistent with the existing literature [9, 39]. Some of these households may have greater constraints that limit their migration duration. For these households, earning lot of remittances is often not a priority as mentioned earlier. They rather prefer longer stays with their families by optimally diversifying their risks at the origin before migrating.”

Also, we have cited the most recent papers (to our knowledge) on destination choices during migration, as our objectives are in this direction.

Comment 2. Neglect of Land Privatization and Land Tenure Insecurity

The manuscript fails to consider the structural impacts of land privatization and tenure insecurity on migration decisions. Since the 1980s, land market privatization in Bangladesh has led to:Land fragmentation; Increasing concentration of land ownership; Expansion of informal tenancy markets; Exclusion of marginal and landless farmers from productive land.

As a result, large numbers of rural poor households are compelled to engage in short-term agricultural labor migration to survive. Migration is not merely a seasonal income strategy, but also a structural outcome of land exclusion and market-driven land reforms.

I recommend:

Including land tenure and land market exclusion as structural factors in the analysis;

Reassessing migration formation mechanisms from the perspective of land privatization and land loss;

Referring to relevant studies on land tenure insecurity and agrarian transformation in South Asia.

Responses: We agree that land ownership affects rural poor’s migration decision, particularly for choosing migration as opposed to non-migration. Similar results are also found in a recent study [Rana et al., 2025].

In our study, we have also controlled for households’ land ownership (captured by the variable “agricultural landholdings”) in all three equations: migration vs. non-migration, temporary vs. longer-term migration, and rural vs. urban destination selections. As shown in Table 4 in the results section (p. 17), land ownership does not significantly affect their choice of destinations. While we agree that agricultural land ownership could affect households’ migration decision, we could not derive any conceptual links between land ownership and destination choice during temporary migration. Accordingly, as our focus is on the latter, we do not elaborate on this variable in the discussion of this paper.

Comment 3. Overly Simplified Classification of Destination Types

The current definition of “rural destinations” is unclear and overly broad. In practice, some destinations categorized as rural may include small towns or peri-urban spaces, reflecting distinct migration patterns.

I recommend:

Clarifying how “rural” is defined in the survey;

Refining the typology of destinations to avoid conflating heterogeneous migration pathways.

Responses: We apologize for this oversight. In the revised version, and on p.6, now we define rural and urban destinations as follows:

“Following the definition provided by the Bangladesh Bureau of Statistics [49], we classify urban destinations as areas located within the jurisdictions of City Corporations, Municipalities, Sub-district Headquarters (not declared as Municipalities), Cantonment areas, and Growth Centres enlisted by the Local Government Division. All other areas are classified as rural destinations. Additionally, we collect information on the type of work undertaken by migrants at their destinations, which we use to further differentiate between rural and urban destinations when migrants are unable to identify the destination type.”

Comment 4. Overly Narrow Measurement of Migration Returns

Migration outcomes are measured solely in terms of household income and remittances. However, for land-constrained households, livelihood diversification and risk management are equally important motivations. I recommend:

Expanding the discussion of non-income-based returns, such as risk mitigation and livelihood security;

Clarifying that income maximization may not be the sole objective of all migration decisions.

Responses: This paper examines income effects only in relation to destination choice during temporary migration; a broader discussion of migration returns lies outside its scope. However, this might be addressed in future research. We write on p.22, “…..Finally, while this study focuses primarily on income effects, future work could also explore non-income-related returns to destination choices.”

For temporary migration, income is often the primary—if not the sole—purpose, as indicated in the literature (Coffey et al., 2014, Khandker and Mahmud, 2012, Mishra, 2016, Rana & Qaim, 2024; Tiwari et al., 2022, Wang et al., 2021). Therefore, in this paper, we focus on this trait of temporary migration while interpreting income as a livelihood diversification strategy.

In this context, we find that rural-bound temporary migration is indeed an important risk diversification strategy for constrained, poor rural households. We write on p. 19-20 the following:

“…. As we have seen from equation (3) (Table A5 in the Online Appendix), farm labor and family obligations are critical factors in choosing temporary over longer-term migration, which is also consistent with the existing literature [9, 39]. Some of these households may have greater constraints that limit their migration duration. For these households, earning lot of remittances is often not a priority as mentioned earlier. They rather prefer longer stays with their families by optimally diversifying their risks at the origin before migrating.

We have also demonstrated in equation (4) (Table 4) that households’ engagement in livestock farming and selling labor at the origin is positively and significantly associated with their choice of rural over urban destinations. Similarly, rural-bound temporary migrants earn significantly more from these two sources (Table A4 in the Online Appendix). This explains the significantly higher income from the local labor market (loc_incj) for rural-bound temporary migrants, as presented in Table 5.

Some of these income options, particularly livestock farming, create significant labor constraints at the origin, prompting shorter-duration temporary migration, mentioned earlier. For such short-duration migration, rural destinations with a more favorable income-to-cost ratio allow migrants to maximize utility through gaining a greater satisfaction from their limited migration time and income. This includes the satisfaction to save their “hard-earned” income [29], balance household labor needs, and reduce time away from home. Conversely, urban destinations may offer higher total income but require longer stays to offset higher living costs, which can lower overall utility for migrants, particularly from households with farm labor or family constraints. In other words, rural-bound temporary migration enables more efficient trade-offs, supporting longer stays with the family and risks diversification at the origin, while urban-bound migration often relies solely on remittances.”

Comment 5. Overly Narrow Policy Recommendations

The current policy recommendations focus primarily on supporting rural-to-rural migration. However, the structural drivers of forced migration, such as land exclusion and agricultural risks, are not addressed.

I recommend:

Proposing policies that mitigate agricultural seasonality and land tenure insecurity;

Supporting diversification of rural employment and strengthening land rights protections;

Developing small-town labor markets as alternative employment spaces;

Framing migration policy within a broader risk management and livelihood improvement strategy.

Responses: We agree on our overly narrow policy recommendations. In the revised version, on p. 22, we write aligning with our main objectives:

“…….. Policy support could include providing wage information, reducing job search costs, and improving inter-district transportation networks.

Moreover, to support distress-driven temporary migrants, promoting early harvesting rice varieties could reduce lean periods. Expanding local economies to support rural livelihoods during these times is also essential.”

----------------------------------------------

Minor Comments

Comment 1) Clarify whether land loss, environmental degradation, or pollution were considered in the survey instrument.

Responses: We controlled “per capita agricultural landholdings” in all models. However, we did not capture land loss in survey instruments.

Pollution was considered as a potential factor for “difficulty for living in cities”, thus incorporated in “Urban Negativity” variable (see Table 1 on p.9).

“Flood vulnerability of villages” is controlled in the model to proxy for environmental degradation.

Comment 2) Specify whether indicators like “income-to-cost ratio” and “physical comfort” were based on subjective perceptions or objective measures.

Response: These were subjective ratings in 1-10 Likert scale as explained on p. 10. We have also specified the “subjective” nature of these data in the revised version.

Comment 3) Ensure consistency in describing the relationship between migration distance and migration costs.

Response: In the revised version, on p.11, we specify: “Migration distance is treated as a proxy for migration costs.”

------------------------------------------------------------------------

Reviewers’ comments copied from the email body:

Review Comments to the Author

Reviewer #1: I have read the article “Destination choices during internal temporary migration: Evidence from northern Bangladesh” with great interest.

Amid growing attention to rural poverty and seasonal hunger, the study is especially important for understanding non-urban destination preferences among poor migrants—an under-researched but relevant topic in migration studies. By focusing on temporary migration in northern Bangladesh, the article challenges prevailing assumptions that urban destinations always offer better economic outcomes. The research addresses empirical gaps by analyzing the utility maximization of rural-bound migrants, drawing implications for policy design targeting rural development and labor mobility.

Specific comments: The methodology employed is suitable for addressing self-selection bias and potential endogeneity, although the model complexity risks over-specification. The choice of instruments and exclusion restrictions is mostly justified (e.g., random economic shocks as an exclusionary variable), yet some relationships (e.g., urban perception and network effects) remain potentially endogenous and could benefit from sensitivity analysis.

Responses: We agree. In the revised version, in Methodology section on p. 11, we write: “….. Furthermore, migrant network (Ni) and urban negativity (Ui) variables may remain potentially endogenous in both models discussed above. To assess their sensitivity, we re-estimate the main model (Eq. 4) excluding these variables sequentially.”

Later in the results section, on p. 18, we interpret the results: “… the main results remain insensitive to potentially endogenous networks and perception variables, as demonstrated in Table A10 in the Online Appendix.”

Also, we remain cautious in interpreting the results, emphasizing on association rather than causality. At the beginning of the results section, on p. 16, we write: “These results should be interpreted as associations rather than causal relationships.”

Specific Comments: The results indicate that while rural destinations offer a better income-to-cost ratio, they do not maximize household income compared to urban migration. This reveals that destination choices are shaped not merely by wages but also by constraints like family obligations, duration

Attachment Submitted filename: Reviewer_responses.docx

# Peer review: Decision Letter 1

**DOI:** 10.1371/journal.pone.0352665.r003

**Authors:** Tianlong You, Tianlong You

15 Apr 2026

PONE-D-25-24890R1

Destination choices during internal temporary migration: Evidence from northern Bangladesh

PLOS One

Dear Dr. Rana,

Thank you for submitting your manuscript to PLOS ONE. After careful consideration, we feel that it has merit but does not fully meet PLOS ONE’s publication criteria as it currently stands. Therefore, we invite you to submit a revised version of the manuscript that addresses the points raised during the review process.

The reviewers have returned their reports. One recommends acceptance of the manuscript, while the other recommends minor revision. In light of these evaluations, I am pleased to invite you to revise the manuscript accordingly and resubmit it as a minor revision.

Please submit your revised manuscript by May 30 2026 11:59PM. If you will need more time than this to complete your revisions, please reply to this message or contact the journal office at plosone@plos.org. When you're ready to submit your revision, log on to https://www.editorialmanager.com/pone/ and select the 'Submissions Needing Revision' folder to locate your manuscript file.

Please include the following items when submitting your revised manuscript:

- A letter that responds to each point raised by the academic editor and reviewer(s). You should upload this letter as a separate file labeled 'Response to Reviewers'.
- A marked-up copy of your manuscript that highlights changes made to the original version. You should upload this as a separate file labeled 'Revised Manuscript with Track Changes'.
- An unmarked version of your revised paper without tracked changes. You should upload this as a separate file labeled 'Manuscript'.

If you would like to make changes to your financial disclosure, please include your updated statement in your cover letter. Guidelines for resubmitting your figure files are available below the reviewer comments at the end of this letter.

If applicable, we recommend that you deposit your laboratory protocols in protocols.io to enhance the reproducibility of your results. Protocols.io assigns your protocol its own identifier (DOI) so that it can be cited independently in the future. For instructions see: https://journals.plos.org/plosone/s/submission-guidelines#loc-laboratory-protocols. Additionally, PLOS ONE offers an option for publishing peer-reviewed Lab Protocol articles, which describe protocols hosted on protocols.io. Read more information on sharing protocols at https://plos.org/protocols?utm_medium=editorial-email&utm_source=authorletters&utm_campaign=protocols.

As the corresponding author, your ORCID iD is verified in the submission system and will appear in the published article. PLOS supports the use of ORCID, and we encourage all coauthors to register for an ORCID iD and use it as well. Please encourage your coauthors to verify their ORCID iD within the submission system before final acceptance, as unverified ORCID iDs will not appear in the published article. *Only*  the individual author can complete the verification step; PLOS staff *cannot*  verify ORCID iDs on behalf of authors.

We look forward to receiving your revised manuscript.

Kind regards,

Tianlong You, Ph.D.

Academic Editor

PLOS One

**Journal Requirements:**

1. If the reviewer comments include a recommendation to cite specific previously published works, please review and evaluate these publications to determine whether they are relevant and should be cited. There is no requirement to cite these works unless the editor has indicated otherwise.

2. Please review your reference list to ensure that it is complete and correct. If you have cited papers that have been retracted, please include the rationale for doing so in the manuscript text, or remove these references and replace them with relevant current references. Any changes to the reference list should be mentioned in the rebuttal letter that accompanies your revised manuscript. If you need to cite a retracted article, indicate the article’s retracted status in the References list and also include a citation and full reference for the retraction notice.

**Additional Editor Comments:**

Dear Authors,

The reviewers have now returned their reports. One recommends acceptance of the manuscript, while the other recommends minor revision.

In light of these evaluations, I am pleased to invite you to revise the manuscript accordingly and resubmit it as a minor revision.

Best regards,

[Note: HTML markup is below. Please do not edit.]

Reviewers' comments:

Reviewer's Responses to Questions

**Comments to the Author**

1. If the authors have adequately addressed your comments raised in a previous round of review and you feel that this manuscript is now acceptable for publication, you may indicate that here to bypass the “Comments to the Author” section, enter your conflict of interest statement in the “Confidential to Editor” section, and submit your "Accept" recommendation.

Reviewer #3: All comments have been addressed

Reviewer #4: All comments have been addressed

**********

2. Is the manuscript technically sound, and do the data support the conclusions?

The manuscript must describe a technically sound piece of scientific research with data that supports the conclusions. Experiments must have been conducted rigorously, with appropriate controls, replication, and sample sizes. The conclusions must be drawn appropriately based on the data presented.

Reviewer #3: Yes

Reviewer #4: Yes

**********

3. Has the statistical analysis been performed appropriately and rigorously?

Reviewer #3: Yes

Reviewer #4: Yes

**********

4. Have the authors made all data underlying the findings in their manuscript fully available?

The PLOS Data policy requires authors to make all data underlying the findings described in their manuscript fully available without restriction, with rare exception (please refer to the Data Availability Statement in the manuscript PDF file). The data should be provided as part of the manuscript or its supporting information, or deposited to a public repository. For example, in addition to summary statistics, the data points behind means, medians and variance measures should be available. If there are restrictions on publicly sharing data—e.g. participant privacy or use of data from a third party—those must be specified.

Reviewer #3: Yes

Reviewer #4: Yes

**********

5. Is the manuscript presented in an intelligible fashion and written in standard English?

PLOS ONE does not copyedit accepted manuscripts, so the language in submitted articles must be clear, correct, and unambiguous. Any typographical or grammatical errors should be corrected at revision, so please note any specific errors here.

Reviewer #3: Yes

Reviewer #4: Yes

**********

6. Review Comments to the Author

Please use the space provided to explain your answers to the questions above. You may also include additional comments for the author, including concerns about dual publication, research ethics, or publication ethics. (Please upload your review as an attachment if it exceeds 20,000 characters)

Reviewer #3: (No Response)

Reviewer #4: I would like to thank the author(s) for their effort in preparing this manuscript. This is a commendable effort by the authors; however, there are some areas that require further refinement before the manuscript is ready for publication. My overall comments on the manuscript are as follows:

• In the Introduction, the research gap needs to be more clearly elaborated, along with an explanation of how the article addresses this gap. The research question is currently missing and should be explicitly stated. Additionally, the article's overall structure should be clearly outlined.

• The article should include a dedicated section on the conceptual framework. This section should provide a brief review of relevant literature and explain how household-level migration decisions are determined.

• In the Materials and Methods section, a map should be included to illustrate the geographical context of the study area. The rationale for selecting these specific locations should also be clearly justified. Additionally, a table should be provided to present the sample distribution by district, upazila, and union. The sampling process—particularly the selection of households—needs to be explained in detail. A separate table should also be included to present the socio-demographic profile of the sample households.

• A Discussion section should be included to interpret the results and explain how they relate to the broader literature. This section should highlight both the similarities with existing studies and the differences from them, along with possible reasons for these variations.

• Citations should be avoided in the Conclusion section. It should begin by briefly stating what the article has addressed. The section should then clearly summarize the study's key findings and explain how these results align with the article’s objectives. Furthermore, the conclusion should highlight how the findings contribute to policy development—not only in Bangladesh but also in broader global contexts. Finally, it should outline potential directions for future research.

**********

7. PLOS authors have the option to publish the peer review history of their article (what does this mean?). If published, this will include your full peer review and any attached files.

If you choose “no”, your identity will remain anonymous but your review may still be made public.

**Do you want your identity to be public for this peer review?**  For information about this choice, including consent withdrawal, please see our Privacy Policy.

Reviewer #3: No

Reviewer #4: No

**********

[NOTE: If reviewer comments were submitted as an attachment file, they will be attached to this email and accessible via the submission site. Please log into your account, locate the manuscript record, and check for the action link "View Attachments". If this link does not appear, there are no attachment files.]

To ensure your figures meet our technical requirements, please review our figure guidelines: https://journals.plos.org/plosone/s/figures

You may also use PLOS’s free figure tool, NAAS, to help you prepare publication quality figures: https://journals.plos.org/plosone/s/figures#loc-tools-for-figure-preparation.

NAAS will assess whether your figures meet our technical requirements by comparing each figure against our figure specifications.

# Author comment: Author response to Decision Letter 2

**DOI:** 10.1371/journal.pone.0352665.r004

12 May 2026

Reviewers' comments:

Review Comments to the Author

Reviewer #3: (No Response)

Reviewer #4: I would like to thank the author(s) for their effort in preparing this manuscript. This is a commendable effort by the authors; however, there are some areas that require further refinement before the manuscript is ready for publication. My overall comments on the manuscript are as follows:

Comment # 1: In the Introduction, the research gap needs to be more clearly elaborated, along with an explanation of how the article addresses this gap. The research question is currently missing and should be explicitly stated. Additionally, the article's overall structure should be clearly outlined.

Response: Thank you for all your feedback. We outline research gaps and our contribution to the literature in row # 41-55 (page 3-4) as the following:

“Several studies have examined destination choices for internal migration, but mainly focusing on permanent or longer-term migration types, which differs in nature from temporary migration [24-28]. To our knowledge, only one study [29] examines destination choices for temporary migration, employing an exploratory qualitative methodology with a relatively small sample size. This study highlights the importance of migrants’ individual characteristics, their networks, and perceptions about destinations in shaping destination choices. Notably, this study finds greater income gain perception for rural relative to urban destination choices, questioning the common assumption that urban destinations offer greater gains.

Yet perceived gains may not necessarily correspond to actual income effects. Destination choices may instead reflect differences in migration costs, remittance patterns, and households’ need to balance migration with origin-based livelihood activities, aspects that remain unclear in the existing literature. By examining both the determinants and income effects of temporary rural versus urban migration, we investigate whether destination choices are primarily driven by realized economic returns or by broader household risk-diversification strategies.

We employ a quantitative methodology to achieve this.……”

We outline our research question in the row # 38-40 (page 3) as below:

“…..This raises a fundamental question as to why do many temporary migrants prefer rural over urban destinations, a topic poorly understood in the existing literature, which we address in this paper.”

The article’s overall structure is outlined in row # 58-61 (page 4) as below:

“We organize this article as follows: Section 2 presents sampling, data, conceptual framework, and models used to achieve our research objectives. The empirical results are presented and discussed in Section 3. Finally, we conclude the article and outline its policy implications in Section 4.”

Comment # 2: The article should include a dedicated section on the conceptual framework. This section should provide a brief review of relevant literature and explain how household-level migration decisions are determined.

Response: For the brevity, we have merged conceptual framework with model specifications. Clarified in the text, row # 162 (page 8). Also, we have included the conceptual framework graph (Figure 2, page 8) and explained it while specifying our models. Existing literature are also discussed in this section while building the model equations and explaining variables (page 9-14).

Comment # 3: In the Materials and Methods section, a map should be included to illustrate the geographical context of the study area. The rationale for selecting these specific locations should also be clearly justified. Additionally, a table should be provided to present the sample distribution by district, upazila, and union. The sampling process—particularly the selection of households—needs to be explained in detail. A separate table should also be included to present the socio-demographic profile of the sample households.

Response: Study area map is included in Figure 1 (page 5). We use a multi-stage sampling procedure to select study areas and collect household data. Rationale for selecting study areas and household sampling procedures is explained in “2.1 Sampling procedure” section, row # 63-104 (page 4-6). Sample distribution is presented in Table A1 in the Online Appendix.

We add a paragraph outlining relevant socio-demographic information of the sampled households in the section “2.3 Socio-demographic and migration data” (row # 129-135, page 7).

Comment # 4: A Discussion section should be included to interpret the results and explain how they relate to the broader literature. This section should highlight both the similarities with existing studies and the differences from them, along with possible reasons for these variations.

Response: For the brevity, we have merged discussions with descriptive and regression results in Section “3. Empirical results and discussions”.

We have highlighted similarities of our findings with the existing literature where necessary, such as Footnote # 6 (page 18), row # 478, 526, and 541.

In the destination choice equation (4), we do not find notable variations from the existing studies. Our income effect equation (5) is novel, and we use its findings and those from equation (4) to clarify destination decision-making process, which is our contribution to the literature. We have clarified our contributions in the Results section as below:

“These findings make a two-fold contribution to the literature: first, they clarify [29]’s ambiguous conclusion about the better income-to-cost ratio characteristic of rural destinations; and second, they shed light on the complex destination decision-making processes during temporary migration, which are often misunderstood from a monetary return’s perspective.” (row # 518-521, page 22).

Then in the conclusions, we summarize our contributions as the following:

“Together, these findings suggest that destination choices during temporary migration reflect broader household risk-diversification and labor allocation strategies rather than simple income maximization.” (row # 551-553, page 23).

We also highlight the similar in the abstract, row # 14-19.

Comment # 5: Citations should be avoided in the Conclusion section. It should begin by briefly stating what the article has addressed. The section should then clearly summarize the study's key findings and explain how these results align with the article’s objectives. Furthermore, the conclusion should highlight how the findings contribute to policy development—not only in Bangladesh but also in broader global contexts. Finally, it should outline potential directions for future research.

Response: Citations have been minimized in the Conclusion section. In the first paragraph, row # 530 to 537 (page 22), we briefly state what our study addresses. In the next two paragraphs, row # 538-553 (page 22-23), we discuss our findings aligning with the research objectives.

Policy implications of the paper are explained in the next three paragraphs, row # 554-572 (page 23). Policy recommendations are made for poor agrarian contexts, exemplifying with Bangladesh, where these recommendations are more contextually relevant.

Future research directions are outlined in the last paragraph, row # 573-578 (page 24).

Attachment Submitted filename: Reviewer feedback.docx

# Peer review: Decision Letter 2

**DOI:** 10.1371/journal.pone.0352665.r005

**Authors:** Tianlong You, Tianlong You, Tianlong You

15 Jun 2026

Destination Choices during Internal Temporary Migration: Evidence from northern Bangladesh

PONE-D-25-24890R2

Dear Dr. Rana,

We’re pleased to inform you that your manuscript has been judged scientifically suitable for publication and will be formally accepted for publication once it meets all outstanding technical requirements.

Within one week, you’ll receive an e-mail detailing the required amendments. When these have been addressed, you’ll receive a formal acceptance letter and your manuscript will be scheduled for publication.

An invoice will be generated when your article is formally accepted. Please note, if your institution has a publishing partnership with PLOS and your article meets the relevant criteria, all or part of your publication costs will be covered. Please make sure your user information is up-to-date by logging into Editorial Manager at Editorial Manager® and clicking the ‘Update My Information' link at the top of the page. For questions related to billing, please contact billing support.

If your institution or institutions have a press office, please notify them about your upcoming paper to help maximize its impact. If they’ll be preparing press materials, please inform our press team as soon as possible -- no later than 48 hours after receiving the formal acceptance. Your manuscript will remain under strict press embargo until 2 pm Eastern Time on the date of publication. For more information, please contact onepress@plos.org.

Kind regards,

Tianlong You, Ph.D.

Academic Editor

PLOS One

Additional Editor Comments (optional):

Reviewers' comments:

Reviewer's Responses to Questions

**Comments to the Author**

1. If the authors have adequately addressed your comments raised in a previous round of review and you feel that this manuscript is now acceptable for publication, you may indicate that here to bypass the “Comments to the Author” section, enter your conflict of interest statement in the “Confidential to Editor” section, and submit your "Accept" recommendation.

Reviewer #3: All comments have been addressed

Reviewer #4: All comments have been addressed

**********

2. Is the manuscript technically sound, and do the data support the conclusions?

The manuscript must describe a technically sound piece of scientific research with data that supports the conclusions. Experiments must have been conducted rigorously, with appropriate controls, replication, and sample sizes. The conclusions must be drawn appropriately based on the data presented.

Reviewer #3: (No Response)

Reviewer #4: Yes

**********

3. Has the statistical analysis been performed appropriately and rigorously?

Reviewer #3: (No Response)

Reviewer #4: Yes

**********

4. Have the authors made all data underlying the findings in their manuscript fully available?

The PLOS Data policy requires authors to make all data underlying the findings described in their manuscript fully available without restriction, with rare exception (please refer to the Data Availability Statement in the manuscript PDF file). The data should be provided as part of the manuscript or its supporting information, or deposited to a public repository. For example, in addition to summary statistics, the data points behind means, medians and variance measures should be available. If there are restrictions on publicly sharing data—e.g. participant privacy or use of data from a third party—those must be specified.

Reviewer #3: (No Response)

Reviewer #4: Yes

**********

5. Is the manuscript presented in an intelligible fashion and written in standard English?

PLOS ONE does not copyedit accepted manuscripts, so the language in submitted articles must be clear, correct, and unambiguous. Any typographical or grammatical errors should be corrected at revision, so please note any specific errors here.

Reviewer #3: (No Response)

Reviewer #4: Yes

**********

6. Review Comments to the Author

Please use the space provided to explain your answers to the questions above. You may also include additional comments for the author, including concerns about dual publication, research ethics, or publication ethics. (Please upload your review as an attachment if it exceeds 20,000 characters)

Reviewer #3: (No Response)

Reviewer #4: I am satisfied that the authors have appropriately addressed the comments and incorporated the necessary revisions into the manuscript.

**********

7. PLOS authors have the option to publish the peer review history of their article (what does this mean?). If published, this will include your full peer review and any attached files.

If you choose “no”, your identity will remain anonymous but your review may still be made public.

**Do you want your identity to be public for this peer review?**  For information about this choice, including consent withdrawal, please see our Privacy Policy.

Reviewer #3: No

Reviewer #4: **Yes:**  Md Zakir Hossain

**********

# Editor report: Acceptance letter

**DOI:** 10.1371/journal.pone.0352665.r006

**Authors:** Tianlong You, Tianlong You, Tianlong You

PONE-D-25-24890R2

PLOS One

Dear Dr. Rana,

I'm pleased to inform you that your manuscript has been deemed suitable for publication in PLOS One. Congratulations! Your manuscript is now being handed over to our production team.

At this stage, our production department will prepare your paper for publication. This includes ensuring the following:

* All references, tables, and figures are properly cited

* All relevant supporting information is included in the manuscript submission,

* There are no issues that prevent the paper from being properly typeset

You will receive further instructions from the production team, including instructions on how to review your proof when it is ready. Please keep in mind that we are working through a large volume of accepted articles, so please give us a few days to review your paper and let you know the next and final steps.

Lastly, if your institution or institutions have a press office, please let them know about your upcoming paper now to help maximize its impact. If they'll be preparing press materials, please inform our press team within the next 48 hours. Your manuscript will remain under strict press embargo until 2 pm Eastern Time on the date of publication. For more information, please contact onepress@plos.org.

You will receive an invoice from PLOS for your publication fee after your manuscript has reached the completed accept phase. If you receive an email requesting payment before acceptance or for any other service, this may be a phishing scheme. Learn how to identify phishing emails and protect your accounts at https://explore.plos.org/phishing.

If we can help with anything else, please email us at customercare@plos.org.

Thank you for submitting your work to PLOS ONE and supporting open access.

Kind regards,

PLOS ONE Editorial Office Staff

on behalf of

Professor Tianlong You

Academic Editor

PLOS One
